# Bioinformatic Analyses of Subgroup-A Members of the Wheat bZIP Transcription Factor Family and Functional Identification of *TabZIP174* Involved in Drought Stress Response

**DOI:** 10.3389/fpls.2016.01643

**Published:** 2016-11-16

**Authors:** Xueyin Li, Biane Feng, Fengjie Zhang, Yimiao Tang, Liping Zhang, Lingjian Ma, Changping Zhao, Shiqing Gao

**Affiliations:** ^1^College of Agronomy, Northwest A & F UniversityYangling, China; ^2^Beijing Municipal Key Laboratory of Molecular Genetics of Hybrid Wheat, Beijing Engineering Research Center for Hybrid Wheat, Beijing Academy of Agriculture and Forestry SciencesBeijing, China; ^3^College of Agriculture, Shanxi Agricultural UniversityTaigu, China

**Keywords:** wheat, bZIP, Subgroup A, expression profile, subcellular localization, *TabZIP174*, transgenic *Arabidopsis*, drought tolerance

## Abstract

Extensive studies in *Arabidopsis* and rice have demonstrated that Subgroup-A members of the bZIP transcription factor family play important roles in plant responses to multiple abiotic stresses. Although common wheat (*Triticum aestivum*) is one of the most widely cultivated and consumed food crops in the world, there are limited investigations into Subgroup A of the bZIP family in wheat. In this study, we performed bioinformatic analyses of the 41 Subgroup-A members of the wheat bZIP family. Phylogenetic and conserved motif analyses showed that most of the Subgroup-A bZIP proteins involved in abiotic stress responses of wheat, *Arabidopsis*, and rice clustered in Clade A1 of the phylogenetic tree, and shared a majority of conserved motifs, suggesting the potential importance of Clade-A1 members in abiotic stress responses. Gene structure analysis showed that *TabZIP* genes with close phylogenetic relationships tended to possess similar exon–intron compositions, and the positions of introns in the hinge regions of the bZIP domains were highly conserved, whereas introns in the leucine zipper regions were at variable positions. Additionally, eleven groups of homologs and two groups of tandem paralogs were also identified in Subgroup A of the wheat bZIP family. Expression profiling analysis indicated that most Subgroup-A *TabZIP* genes were responsive to abscisic acid and various abiotic stress treatments. TabZIP27, TabZIP74, TabZIP138, and TabZIP174 proteins were localized in the nucleus of wheat protoplasts, whereas TabZIP9-GFP fusion protein was simultaneously present in the nucleus, cytoplasm, and cell membrane. Transgenic *Arabidopsis* overexpressing *TabZIP174* displayed increased seed germination rates and primary root lengths under drought treatments. Overexpression of *TabZIP174* in transgenic *Arabidopsis* conferred enhanced drought tolerance, and transgenic plants exhibited lower water loss rates, higher survival rates, higher proline, soluble sugar, and leaf chlorophyll contents, as well as more stable osmotic potential under drought conditions. Additionally, overexpression of *TabZIP174* increased the expression of stress-responsive genes (*RD29A, RD29B, RAB18, DREB2A, COR15A*, and *COR47*). The improved drought resistance might be attributed to the increased osmotic adjustment capacity. Our results indicate that TabZIP174 may participate in regulating plant response to drought stress and holds great potential for genetic improvement of abiotic stress tolerance in crops.

## Introduction

Plants often undergo adverse environmental stresses, such as drought, high salinity, and low temperature, which hinder plant growth and development, and decrease grain yield. Plants have evolved diverse regulatory mechanisms at the biochemical, cellular, physiological, and morphological levels to cope with these unfavorable environmental conditions (Zhu, 2002; Hirayama and Shinozaki, 2010; Krasensky and Jonak, 2012; Zhang et al., 2015).

During the response and adaptation to various abiotic stresses, transcription factors act as triggers of gene expression and play important regulatory roles (Xiang et al., 2008). The basic leucine zipper (bZIP) proteins make up one of the largest and most diverse transcription factor families in plants, and regulate various biological processes, including seed maturation and germination, photomorphogenesis, floral induction and development, and pathogen defense (Jakoby et al., 2002; Nijhawan et al., 2008). The bZIP proteins possess an eponymous bZIP domain, which is comprised of a basic region and a leucine zipper (Hurst, 1994). The basic region is highly conserved and is composed of approximately 16 amino acid (aa) residues, including an invariant N-X_7_-R/K motif. The basic region takes charge of sequence-specific DNA binding. By comparison, the leucine zipper is less conserved and comprises heptad repeats of leucine (Leu) or other bulky hydrophobic amino acid (such as Ile, Val, Phe, or Met) residues positioned accurately nine amino acid residues toward the C-terminus to create an amphipathic helix that confers homo- or hetero-dimerization specificity (Jakoby et al., 2002; Nijhawan et al., 2008; Li X. et al., 2015).

Members of the bZIP transcription factor family were comprehensively identified or predicted in some plant genomes, such as *Arabidopsis*, rice, soybean, sorghum, maize, cucumber, grapevine, castor bean, *Brachypodium distachyon*, tomato, wheat, *Triticum urartu, Aegilops tauschii*, barley, and apple (Jakoby et al., 2002; Liao et al., 2008; Nijhawan et al., 2008; Wang et al., 2011; Wei et al., 2012; Baloglu et al., 2014; Jin et al., 2014; Liu et al., 2014; Li D. et al., 2015; Li X. et al., 2015; Liu and Chu, 2015; Zhao et al., 2016). bZIP family members were classified into 14 subgroups (Jakoby et al., 2002; Li X. et al., 2015). Extensive studies in *Arabidopsis* and rice demonstrated that Subgroup-A proteins of the bZIP family participated in mediating abscisic acid (ABA) signaling and/or regulating abiotic stress responses in plants, and held great potential in enhancing the resistance of transgenic plants to multiple abiotic stresses. In *Arabidopsis*, Subgroup-A members of the bZIP family were designated as ABFs (ABA-responsive element binding factors), AREBs (ABA response element binding factors), or DPBFs (*Dc3* promoter binding factors) in different studies (Kim S. Y. et al., 1997; Choi et al., 2000; Uno et al., 2000; Kim et al., 2002). However, several of these independently identified loci were identical. Most AtbZIPs in Subgroup A were characterized in previous studies, including AtbZIP12/DPBF4/EEL, AtbZIP35/ABF1, AtbZIP36/ABF2/AREB1, AtbZIP37/ABF3/DPBF5, AtbZIP38/ABF4/AREB2, AtbZIP39/ABI5/DPBF1, AtbZIP40/GBF4, AtbZIP66/AREB3/DPBF3, and AtbZIP67/DPBF2 (Kim J. et al., 1997; Choi et al., 2000; Bensmihen et al., 2002; Jakoby et al., 2002; Kim et al., 2002). The AREBs/ABFs were important transcription factors involved in response to ABA and various abiotic stresses, including drought and salinity (Kobayashi et al., 2008). Expression of *ABF* genes was induced by ABA and various abiotic stress treatments, and as master transcription factors, ABF2, ABF3, and ABF4 cooperatively mediated drought-responsive ABA signaling (Choi et al., 2000; Yoshida et al., 2010). *ABF2* overexpression affected multiple stress tolerance and ABA sensitivity of transgenic *Arabidopsis* (Kim et al., 2004). Overexpression of *ABF3* or *ABF4* reduced transpiration, enhanced drought tolerance and resulted in ABA hypersensitivity in transgenic *Arabidopsis*. However, the *abf3* and *abf4* mutants were defective in dehydration, salt, and ABA responses (Kang et al., 2002; Kim et al., 2004). In rice (*Oryza sativa*), overexpression of *OsbZIP23* (LOC_Os02g52780) significantly increased ABA sensitivity and resistance to drought and high-salinity stresses. The mutation of *OsbZIP23* led to decreased ABA sensitivity and decreased tolerance to drought and salinity stresses; however, this phenotype could be restored after transforming *OsbZIP23* back into the mutant (Nijhawan et al., 2008; Xiang et al., 2008). OsbZIP46 (OsABF2, LOC_Os06g10880) and OsbZIP72 (LOC_Os09g28310) proteins participated in the positive regulation of ABA response and drought resistance of rice (Nijhawan et al., 2008; Lu et al., 2009; Hossain et al., 2010a; Tang et al., 2012). Mutation of *OsABF1* (*OsbZIP12*, LOC_Os01g64730) led to increased sensitivity to salinity and drought stress treatments, and suppressed the expression of ABA- and stress-responsive genes (Nijhawan et al., 2008; Hossain et al., 2010b). Additionally, a recent study revealed that OsABF1 might act as a drought-induced suppressor of floral transition (Zhang et al., 2016). *OsABI5* (*OsbZIP10*, LOC_Os01g64000) was closely related to stress responses of rice (Nijhawan et al., 2008; Zou et al., 2008).

Bread wheat (*Triticum aestivum*; 2*n* = 6*x* = 42; AABBDD) is one of the most widely cultivated and consumed food crops in the world (Jia et al., 2013; Ling et al., 2013). Great efforts have been made worldwide to sequence and annotate the complex hexaploid wheat genome (Brenchley et al., 2012; Jia et al., 2013; Ling et al., 2013; Luo et al., 2013; Choulet et al., 2014; Marcussen et al., 2014; Pfeifer et al., 2014), and recently, a draft sequence of the 17 Gb hexaploid wheat genome has been generated by sequencing isolated chromosome arms (IWGSC, 2014). However, compared with *Arabidopsis* and rice, there were fewer investigations into Subgroup A of the bZIP family in wheat. The wheat *WABI5* gene was orthologous to barley *HvABI5*, and overexpression of *WABI5* in transgenic tobacco plants greatly improved the tolerance to osmotic and freezing stresses, and resulted in a hypersensitivity to ABA at the seedling stage (Kobayashi et al., 2008). A novel *ABI*-like bZIP transcription factor gene, *TaABL1*, was cloned from wheat in our previous study (Xu et al., 2014). The gene *TabZIP60* shared high similarity in protein sequence with *TaABL1*. The expressions of both *TaABL1* and *TabZIP60* were strongly induced by drought, salt, low temperature, and exogenous ABA treatments. Transgenic plants overexpressing *TaABL1* or *TabZIP60* exhibited improved tolerance to multiple abiotic stresses and increased ABA sensitivity (Xu et al., 2014; Zhang et al., 2015). Furthermore, *TaABL1* overexpression hastened stomatal closure of transgenic plants under abiotic stress conditions (Xu et al., 2014). Numerous studies in *Arabidopsis* and rice showed that Subgroup-A *bZIP* genes played vital roles in regulating abiotic stress responses of plants. However, more wheat *bZIP* genes involved in abiotic stress responses remain to be identified from Subgroup A.

Members of the bZIP family in wheat, including most of Subgroup-A members, were identified in our previous study (Li X. et al., 2015). In the present study, we first added a few more bZIP members to Subgroup A. And we report the bioinformatic analyses of Subgroup-A members of the wheat bZIP family. Phylogenetic and conserved motif analyses were performed to reveal the similarities among Subgroup-A bZIP proteins from wheat, *Arabidopsis*, and rice, which suggests the potential importance of Clade-A1 bZIP proteins in abiotic stress response. Gene structures were analyzed to obtain a deeper insight into the exon–intron compositions of Subgroup-A *TabZIP* genes, as well as intron positions within the bZIP domains. Additionally, we also identified homologous *TabZIP* genes within Subgroup A of the wheat bZIP family. Gene expression analysis was performed to characterize the expression profiles of Subgroup-A *TabZIP* genes in response to ABA and multiple abiotic stresses. Subsequently, the subcellular localizations of several TabZIP proteins were confirmed. Functional analysis of the gene *TabZIP174* was carried out by investigating the drought stress tolerance of transgenic *Arabidopsis* plants overexpressing *TabZIP174*, and meanwhile, their physiological traits were monitored. The expression of several stress-responsive genes was also detected by quantitative real-time PCR (qRT-PCR). Our results indicated that TabZIP174 might participate in regulating the response to drought stress. Finally, we proposed the putative mechanism by which TabZIP174 enhanced the drought tolerance of transgenic *Arabidopsis* plants.

## Materials and methods

### Database search for full-length coding sequences

In our previous study, 187 bZIP transcription factor family members were identified from the wheat genome (Li X. et al., 2015). Among these, 35 novel TabZIP proteins belonged to Subgroup A of the wheat bZIP family. To obtain the full-length coding sequences (CDSs) of these Subgroup-A *TabZIP* genes, their CDSs were used as queries to perform similarity search in the Triticeae full-length CDS database (TriFLDB; http://trifldb.psc.riken.jp/v3/index.pl; Mochida et al., 2009).

### Phylogenetic and conserved protein motif analyses

Multiple protein sequence alignment of the Subgroup-A members of the bZIP family in wheat, *Arabidopsis* and rice, including 41 TabZIP, 13 AtbZIP, and 17 OsbZIP proteins, respectively, was performed using the ClustalX program (Version 2.1) with the default settings (Larkin et al., 2007). An unrooted phylogenetic tree was constructed with the neighbor-joining method using MEGA5.0 software (Tamura et al., 2011).

Protein sequences of TabZIPs, AtbZIPs, and OsbZIPs were searched for common conserved motifs using the online tool MEME (Version 4.10.2, http://meme-suite.org/tools/meme; Bailey et al., 2009), and the number of motifs was specified with all other parameters set to default.

### Gene structure analysis

GSDS V2.0 (http://gsds.cbi.pku.edu.cn/; Hu et al., 2015) was used to align the CDSs of the Subgroup-A *TabZIP* genes with their corresponding genomic sequences to analyze their exon–intron structures.

To further analyze the intron-position patterns within the bZIP domains, protein sequences of the bZIP domains of the Subgroup-A TabZIP proteins were extracted using ScanProsite (http://prosite.expasy.org/; de Castro et al., 2006).

### Identification of homologous genes

To identify homologous genes within Subgroup A of the wheat bZIP family, pairwise protein sequence alignments were performed using the BLASTP 2.3.0+ program (Altschul et al., 2005). A pair of homologous genes was defined based on the following criteria: (1) the FASTA-aligned region between their protein sequences covered ≥70% of the longer sequence and (2) the identity of the aligned region was ≥75% (Gu et al., 2002).

### Plant materials and abiotic stress treatments

The wheat (*T. aestivum* L.) genotype “Luohan 9769” was used in this study. After sterilization with 75% ethanol and washing with sterilized water, wheat seeds were germinated and cultivated with double-distilled water in a growth chamber (25°C, 500 μmol/m^2^/s, 12/12 h light/dark photoperiod). Twelve-day-old wheat seedlings were treated with 15% (w/v) polyethylene glycol-6000 (PEG-6000), 250 mM NaCl, 200 μM ABA, or low temperature (4°C). The treated plants were stressed in the PEG or NaCl solutions for 24 h, cultivated under low temperature conditions for 24 h or sprayed once with the ABA solution. Three entire wheat seedlings were harvested as a pooled sample at 0, 1, 3, 6, 12, and 24 h after treatment, respectively.

To investigate the tissue-specific expression pattern of the gene *TabZIP174* in wheat, roots, and leaves of 12-day-old seedlings, as well as roots, stems, leaves, and anthers at the blooming stage, were harvested. At harvest, plant materials were frozen immediately in liquid nitrogen and stored at −80°C.

### RNA extraction and qRT-PCR

Total RNA extraction and first-strand cDNA synthesis were performed as described previously (Li X. et al., 2015). The qRT-PCR analysis was performed using an Eco Real-Time PCR system (Illumina, San Diego, CA, USA) with TaKaRa SYBR® Premix Ex Taq™ (Tli RNaseH Plus) (TaKaRa, Dalian, China). The wheat *Actin* (Gene ID: 542814) and *Arabidopsis Actin2* (AT3G18780) were used as the internal controls for the expression analysis of *TabZIP* genes in wheat and stress-responsive genes in *Arabidopsis*, respectively. The gene-specific primers were designed using Primer Premier 5.0 (Singh et al., 1998). Each reaction was performed in triplicate in a reaction volume of 10 μl as described previously (Li X. et al., 2015). The expression analysis was conducted using the same PCR procedure or with a slightly adjusted annealing temperature. The relative gene expression levels were calculated according to the 2^−ΔΔC_T_^ method (Livak and Schmittgen, 2001).

### Subcellular localization in wheat protoplasts

Nuclear localization signal (NLS) prediction for the Subgroup-A TabZIP proteins was performed with the online tool NucPred (http://www.sbc.su.se/~maccallr/nucpred/; Brameier et al., 2007). To further examine the subcellular localization of the Subgroup-A TabZIP proteins, green fluorescent protein (GFP) expression vectors (*CaMV35S-GFP-NOS*) were constructed. The coding regions of *TabZIP9, TabZIP27, TabZIP74, TabZIP138*, and *TabZIP174* were amplified by PCR with gene-specific primers and independently fused to the N-terminus of GFP in the expression vector. Wheat protoplasts were isolated from the mesophyll tissue of 2-week-old wheat seedlings, and then transformed using the PEG transfection method separately with the plasmid DNA of *35S::TabZIP9-GFP, 35S::TabZIP27-GFP, 35S::TabZIP74-GFP, 35S::TabZIP138-GFP, 35S::TabZIP174-GFP*, and *35S::GFP* control as described previously (Shan et al., 2014). After PEG transfection, wheat protoplasts were incubated in W5 solution (2 mM MES, 154 mM NaCl, 125 mM CaCl_2_, and 5 mM KCl, pH = 5.7) in a dark chamber at 23°C for 18 h, and GFP fluorescence was monitored under a laser-scanning confocal microscope (A1, Nikon Corporation, Tokyo, Japan).

### Generation of transgenic *Arabidopsis*

The full-length opening reading frame of *TabZIP174* was amplified from wheat cDNA with gene-specific primers (forward: 5′-ATGGAGATGCCGGGAGGGA-3′; reverse: 5′-CTACCACGGACCCGTCAGAGTTC-3′), and cloned into the pBI121 vector driven by cauliflower mosaic virus (CaMV) 35S promoter to construct the recombinant vector (*35S::TabZIP174*). The recombinant vector (*35S::TabZIP174*) was introduced into *Agrobacterium tumefaciens* and transformed into *Arabidopsis* (ecotype Columbia-4) using the floral dip method (Zhang et al., 2006). Positive transgenic lines were screened on Murashige and Skoog (MS) medium containing 50 μg/ml kanamycin, and then confirmed by reverse transcription PCR. Independent T_3_-generation transgenic *Arabidopsis* lines with relatively higher *TabZIP174* transcript levels were chosen for further analyses.

### Germination and primary root growth assays

Homozygous T_3_ seeds of *TabZIP174* transgenic lines were used for germination and primary root growth assays. Homozygous T_3_ transgenic and wild type (WT) seeds were surface-sterilized, kept at 4°C in the dark for 3 days and then sown on ½ MS medium solidified with 1.0% (w/v) agar. For the germination assay, the seeds of transgenic lines and WT were placed on ½ MS medium containing no or 5% (w/v) PEG-6000. The germination percentages were calculated daily for 7 days. For the primary root growth assay, 3-day-old seedlings grown on ½ MS medium were transferred to ½ MS medium supplemented with or without 5% PEG-6000, and grown vertically for 4 days prior to measuring primary root lengths.

### Drought tolerance assay

Homozygous T_3_-generation *TabZIP174* transgenic lines were used in the drought tolerance assay. Both transgenic and WT seeds were kept at 4°C in the dark for 3 days and then germinated in a soil mixture (1:1 of vermiculite:humus) in a greenhouse (22°C, relative humidity 70%, and 12/12 h light/dark photoperiod). Two-week-old seedlings were transferred to identical rectangular pots filled with the soil mixture and were regularly watered for two weeks. Subsequently, transgenic and WT plants were cultivated without watering for 4 weeks, and were then rewatered. Survival rates were calculated at 4 days after rewatering. The drought tolerance experiment was carried out in triplicate.

### Physiological characterization of transgenic *Arabidopsis*

To determine the water loss rate under water deficit conditions, the aboveground parts were excised from 4-week-old transgenic and WT plants, and weighted immediately (fresh weight, FW). The samples were then placed on the laboratory bench (22–24°C, relative humidity 40–45%) and weighted at the designated time-points. The samples were finally oven dried at 80°C for 24 h to a constant dry weight (DW) (Mao et al., 2012). The percentages of water loss were measured relative to the initial water contents. Subsequently, relative water contents (RWCs) before oven drying were also calculated. Ten plants for each of transgenic lines and WT were used in this assay.

*Arabidopsis* leaves were harvested at designated time-points during drought treatment and used to measure the free proline content, total soluble sugar content, and osmotic potential. To maximize the sample uniformity at each time-point, leaves of the same size and location were detached from transgenic and WT plants. The free proline content was determined as described previously (Bates et al., 1973). Samples (~0.1 g) were homogenized in 3% sulfosalicylic acid and boiled for 10 min. After the reaction between proline and acid ninhydrin, the absorbance of sample solutions was measured at 520 nm with a UV-Vis spectrophotometer (NanoDrop 2000c, Thermo Scientific, Wilmington, DE, USA). The total soluble sugar content was measured using the anthrone colorimetric method (Yemm and Willis, 1954). Soluble sugars were extracted from homogenized samples (~0.1 g). After the reaction of soluble sugars with anthrone and concentrated sulfuric acid, the absorbance of sample solutions was measured at 620 nm. The proline and soluble sugar contents were determined using their respective standard curves and calculated based on fresh weights. Osmotic potential was measured with an automatic freezing-point osmometer (Multi-OSMETTE™, Model 2430E, Precision Systems Inc., Natick, MA, USA). Ten leaves per *Arabidopsis* line were harvested as a pooled sample and transferred to an injection syringe, and the liquid was then squeezed out of the leaves. The supernatant tissue sap was collected after centrifugation at 13,400 × g for 1 min at room temperature, and then filtered with a filtration column. The filtrate was loaded onto the osmometer to measure the osmotic concentration according to the manufacturer's instructions. The osmotic concentration was measured three times for each sample. Subsequently, the osmotic potential was calculated from the osmotic concentration using the Van't Hoff equation. All of these measurements were repeated three times.

The total chlorophyll content was determined with a portable chlorophyll meter (SPAD-502Plus, Konica Minolta Inc., Tokyo, Japan). Thirty leaves of similar size were selected from each *Arabidopsis* line and used for the *in situ* measurement of the chlorophyll content at each designated time-point during the drought treatment. To minimize the measurement deviations of different leaves, the chlorophyll meter was applied to the same position on the leaves.

## Results

### Phylogenetic and conserved motif analyses of subgroup-A members in the bZIP family

In our previous study, 187 wheat bZIP transcription factor genes were identified from the wheat genome and named based on their chromosomal locations (Li X. et al., 2015). And 35 novel *TabZIP*, 13 *AtbZIP*, and 17 *OsbZIP* genes were identified as Subgroup-A members of the bZIP family based on the phylogenetic analysis (Li X. et al., 2015). To obtain the full-length CDSs of the 35 Subgroup-A *TabZIP* genes, the Triticeae full-length CDS database (TriFLDB) (Mochida et al., 2009) was used to perform similarity search with the CDSs of these 35 *TabZIP* genes as queries. The basic information on these 35 *TabZIP* genes is shown in Table 1. In addition to these 35 novel *TabZIP* genes, Subgroup A of the wheat bZIP family included *TaABF1, TaABI5, WABI5* (*WABI5-1, WABI5-2*, and *WABI5-3*), *TaABL1*, and *TabZIP60* (*TabZIP60-A, TabZIP60-B*, and *TabZIP60-D*) (Johnson et al., 2002, 2008; Kobayashi et al., 2008; Rikiishi et al., 2010; Harris et al., 2013; Xu et al., 2014; Zhang et al., 2015). Therein, *TaABF1, WABI5-3*, and *TabZIP60-A* were identical to *TabZIP23, TabZIP162*, and *TabZIP51* in our nomenclature, respectively. In conclusion, a total of 41 Subgroup-A members of the bZIP family were identified from the wheat genome.

**Table 1:** **Basic information for the Subgroup-A ***TabZIP*** genes**.

***TabZIP***	**Gene ID**	**CDS(nt)**	**PEP(aa)**	**bZIP domain^a^**	**Chromosomal location**	**Precise position on the chromosome**
*TabZIP5*	Traes_1AL_1FFBFB058.1	987	328	256–301	1AL	220288133–220292420
*TabZIP9*	Traes_2AS_3A8C0BD60.1	276	91	1–38	2AS	15377631–15378760
*TabZIP14*	Traes_2AL_3D7807781.1	396	131	54–110	2AL	221472324–221473242
*TabZIP20*	Traes_3AL_58F294736.2	381	126	57–102	3AL	Scaffold IWGSC_CSS_3AL_scaff_3842941: 1–824
*TabZIP23*	Traes_3AL_FC5523394.2	1176	391	305–350	3AL	158927528–158928568
*TabZIP27*	Traes_4AS_F9C171219.1	678	225	153–197	4AS	86640961–86645921
*TabZIP40*	Traes_5AL_79E6A58E6.1	213	70	1–43	5AL	Scaffold IWGSC_CSS_5AL_scaff_2772028: 6–1337
*TabZIP51/TabZIP60-A*	Traes_6AL_E8CD2C02B.1/KJ806558 (GenBank)	1086	361	279–328	6AL	193103591–193107395
*TabZIP54*	Traes_7AL_3ED7A9663.1	255	84	12–68	7AL	Scaffold IWGSC_CSS_7AL_scaff_4376338: 4753–7569
*TabZIP65*	Traes_1BL_DE2CF9613.1	981	326	254–299	1BL	Scaffold IWGSC_CSS_1BL_scaff_3907952: 2851–6847
*TabZIP68*	Traes_2BS_84FB90D88.1	276	91	1–38	2BS	Scaffold IWGSC_CSS_2BS_scaff_5141107: 3–1150
*TabZIP73*	Traes_2BL_709E67A02.1	528	176	104–149	2BL	333582239–333582768
*TabZIP74*	Traes_2BL_94E5996F7.1	405	134	57–113	2BL	300904368–300905361
*TabZIP76*	Traes_2BL_D0D6F6846.1	552	183	93–138	2BL	302757846–302758982
*TabZIP77*	Traes_3B_1F253F060.1	273	90	8–72	3B	629627371–629627608
*TabZIP80*	Traes_3B_3A8224218.2	1176	391	305–350	3B	–
*TabZIP83*	Traes_3B_5D3F7382A.1	1023	340	271–316	3B	–
*TabZIP84*	Traes_3B_6B26CF136.1	858	286	245–286	3B	–
*TabZIP86*	Traes_3B_A796206A0.2	1164	387	305–350	3B	8982474–8985806
*TabZIP90*	Traes_4BL_4C9A415F3.1	663	220	148–192	4BL	58406356–58410423
*TabZIP111*	Traes_5BL_DE53199D3.3	1098	365	284–333	5BL	144705569–144709291
*TabZIP114*	Traes_5BL_FB4EDEA83.2	972	323	251–296	5BL	Scaffold IWGSC_CSS_5BL_scaff_10903754: 7912–12081
*TabZIP127*	Traes_1DL_D9BA83221.2	1107	368	254–299	1DL	106298159–106302141
*TabZIP128*	Traes_1DL_DA67871B9.1	762	253	171–235	1DL	86554441–86556883
*TabZIP133*	Traes_2DL_1F0CDB1CE.1	396	131	54–110	2DL	133012955–133013459
*TabZIP134*	Traes_2DL_1F0CDB1CE1.1	396	131	54–110	2DL	133008772–133009276
*TabZIP135*	Traes_2DL_5610BA574.1	453	151	77–122	2DL	149025389–149026423
*TabZIP138*	Traes_3DL_20ED2EA4C.1	1014	337	265–310	3DL	85255062–85258465
*TabZIP141*	Traes_3DL_E21790878.1	630	209	90–135	3DL	90533952–90535136
*TabZIP148*	Traes_4DL_F38ED7FB6.1	672	223	151–195	4DL	38815726–38820767
*TabZIP158*	Traes_5DL_73CE92096.2	972	323	251–296	5DL	Scaffold IWGSC_CSS_5DL_scaff_2974210: 869–5254
*TabZIP162/WABI5-3*	Traes_5DL_895AA6D35.1/AB362820 (GenBank)	1059	352	272–321	5DL	77875198–77878011
*TabZIP171*	Traes_6DL_F7015CE89.2	525	175	91–136	6DL	157536269–157540353
*TabZIP174*	Traes_7DS_C6A3C10A6.1	1023	340	258–303	7DS	Scaffold IWGSC_CSS_7DS_scaff_3962964: 4–4775
*TabZIP182*	Traes_7DL_EECCC4DBF.1	495	164	92–148	7DL	234881263–234884437
*TabZIP60-B*	KJ806559 (GenBank)	1059	352	270–319	6B	–
*TabZIP60-D*	KJ806560 (GenBank)	1086	361	279–328	6D	–
*WABI5-1*	AB362818 (GenBank)	1068	355	274–323	5A (or 5B)	–
*WABI5-2*	AB362819 (GenBank)	1059	352	272–321	5B (or 5A)	–
*TaABI5*	AB238934 (GenBank)	1173	390	304–349	–	–
*TaABL1*	–	1083	360	279–328	–	–

a *The column entitled “bZIP domain” indicates the positions of the bZIP domains in the protein sequences*.

**Figure 1:**
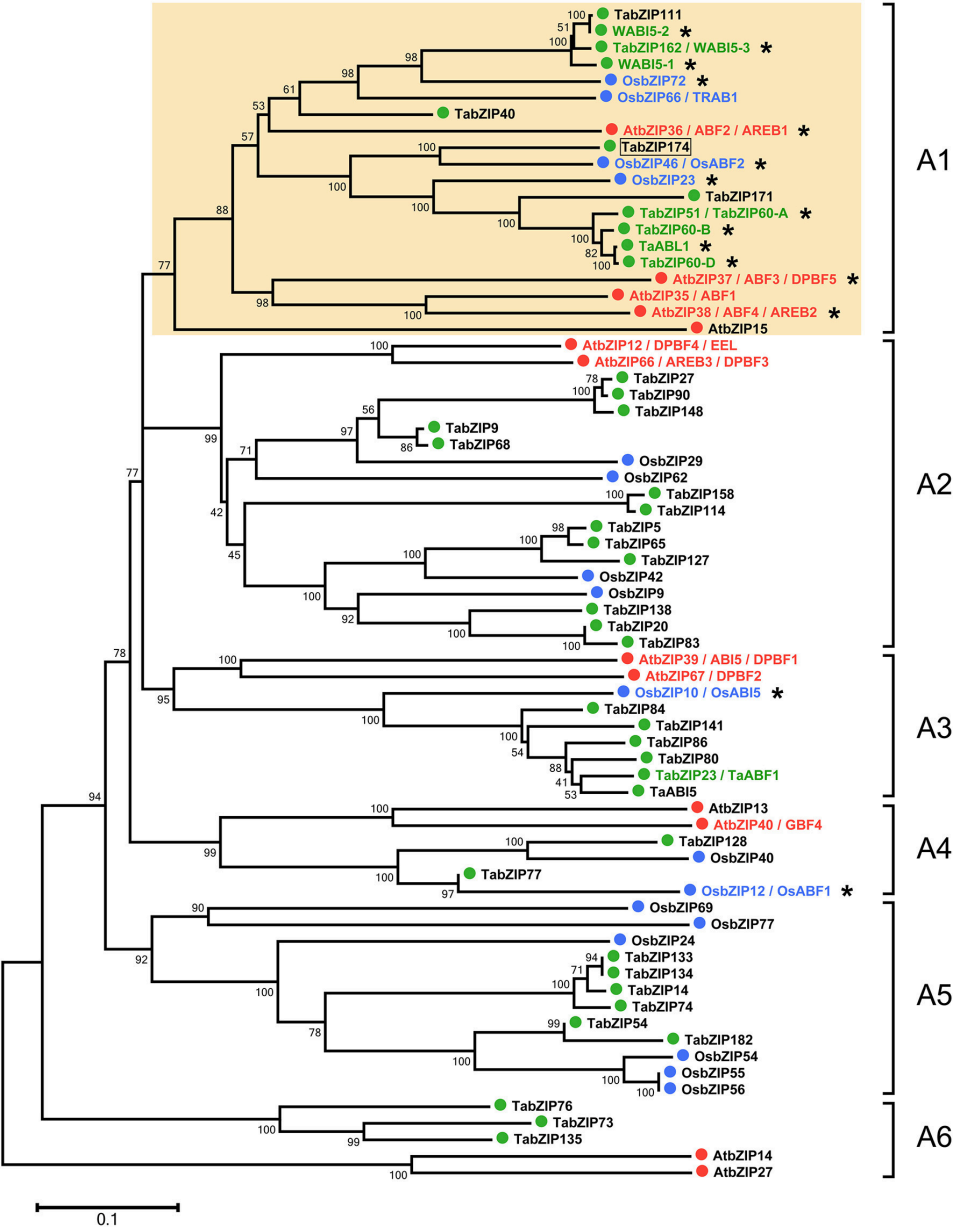
**Phylogenetic analysis of Subgroup-A members of bZIP transcription factor family in wheat, ***Arabidopsis***, and rice**. The phylogenetic analysis was performed using the protein sequences of Subgroup-A members (including 41 TabZIPs, 13 AtbZIPs, and 17 OsbZIPs) in the bZIP family. The unrooted phylogenetic tree was generated by the neighbor-joining method using MEGA5.0 software. All of these Subgroup-A members are further classified into six clades (A1–A6). Numbers above or below branches of the tree indicate bootstrap values. TabZIPs, AtbZIPs, and OsbZIPs are indicated by green, red, and blue circles, respectively, and if they had been characterized in previous studies, their names are colored with green, red, or blue, respectively. Those that had been reported to play important roles in abiotic stress responses are emphasized with asterisks beside their names.

Multiple protein sequence alignment of the Subgroup-A members of the bZIP family in wheat, *Arabidopsis*, and rice (including 41 TabZIP, 13 AtbZIP, and 17 OsbZIP proteins) was performed, and then, an unrooted phylogenetic tree was constructed (Figure 1). A majority (9/13) of the AtbZIPs in the phylogenetic tree had been characterized in previous studies (Menkens and Cashmore, 1994; Choi et al., 2000; Finkelstein and Lynch, 2000; Uno et al., 2000; Lopez-Molina et al., 2001; Bensmihen et al., 2002, 2005; Carles et al., 2002; Kang et al., 2002; Kim et al., 2002, 2004; Lopez-Molina et al., 2002; Finkelstein et al., 2005; Yoshida et al., 2010; Mendes et al., 2013), and three of the nine AtbZIPs played important roles in regulating abiotic stress resistance in *Arabidopsis* (Choi et al., 2000; Kang et al., 2002; Kim et al., 2004; Finkelstein et al., 2005). Additionally, 5 of the 17 OsbZIPs in the phylogenetic tree had important functions in enhancing tolerance of transgenic rice to multiple abiotic stresses (Xiang et al., 2008; Zou et al., 2008; Lu et al., 2009; Hossain et al., 2010a,b; Tang et al., 2012). Among the 41 TabZIPs of Subgroup A, only WABI5-1, TaABL1, and TabZIP60 were involved in regulating plant responses to abiotic stress (Kobayashi et al., 2008; Xu et al., 2014; Zhang et al., 2015). Clade A1 in the phylogenetic tree comprises 20 members, of which 75% (15/20) were functionally characterized, with the ABFs (ABF1–4) among this subset of proteins. On the other hand, most (13/15) of the bZIP proteins previously known to participate in the regulation of abiotic stress responses clustered in Clade A1, indicating that the four other unknown wheat bZIP proteins in Clade A1 (TabZIP40, TabZIP111, TabZIP171, and TabZIP174) were more likely associated with abiotic stress responses.

**Figure 2:**
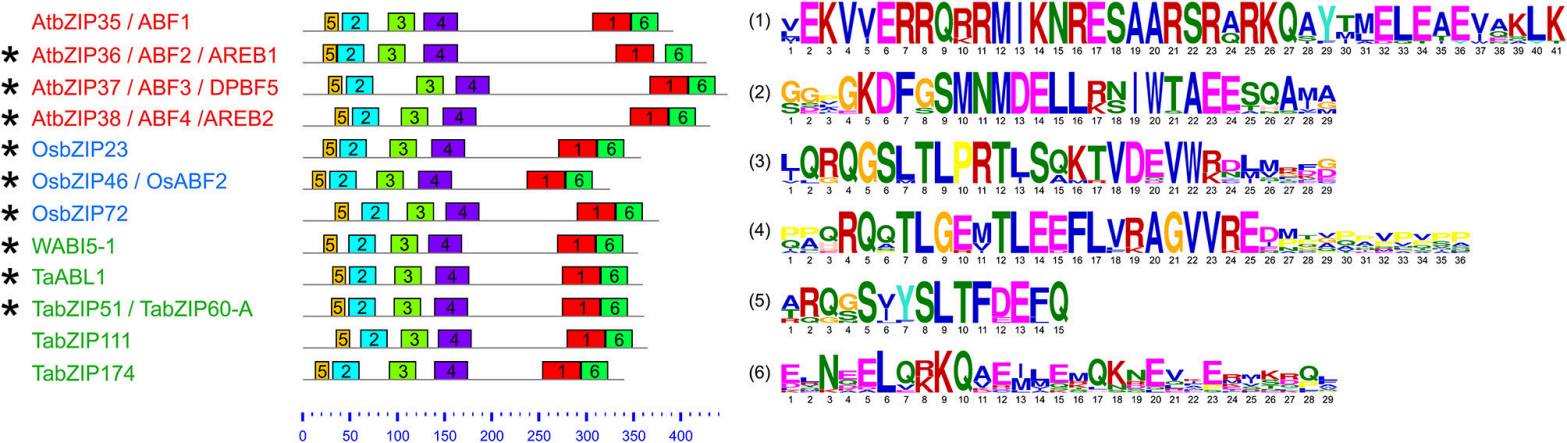
**Distribution and protein sequences of common conserved motifs of Subgroup-A bZIP proteins in wheat, ***Arabidopsis*** and rice**. All of the conserved motifs were identified using MEME with complete protein sequences, and subsequently, the conserved motif alignment was performed by MAST. The green, red, and blue names on the left represent the bZIP proteins from wheat, *Arabidopsis*, and rice, respectively. The bZIP proteins involved in abiotic stress responses are indicated with asterisks beside their names. Motif size is indicated at the bottom of the figure and different motifs are highlighted in different colors (Motif 1 represents the bZIP domain, Motif 5 and 2 compose the conserved domain C1, and Motif 3 and 4 contain the conserved domains C2 and C3, respectively. The conserved domains C1, C2, and C3 contain potential phosphorylation sites). The protein sequences of all of the conserved motifs are shown on the right, and the lengths of these protein sequences are indicated below the corresponding protein sequences. TabZIP111 and TabZIP174 share six conserved motifs with these functionally characterized bZIP proteins, and the protein sequence of each motif is highly conserved among this subset of proteins.

Common conserved motifs in the Subgroup-A proteins of the bZIP family in wheat, *Arabidopsis* and rice were also analyzed (Supplemental Figure 1). Most (9/11) of the Subgroup-A bZIP proteins related to abiotic stress responses (namely, AtbZIP36, AtbZIP37, AtbZIP38, OsbZIP23, OsbZIP46, OsbZIP72, WABI5-1, TaABL1, and TabZIP60) shared a majority of conserved motifs (Supplemental Figure 1). And, notably, TabZIP111 and TabZIP174 shared most of the conserved motifs with the above-mentioned bZIP proteins. Therefore, protein sequence similarity of this subset of proteins, which shared most of the conserved motifs, was further analyzed (Figure 2). These 12 Subgroup-A bZIP proteins had six conserved motifs in common, and the protein sequence of each motif was highly conserved among these bZIP proteins (Figure 2). Nine of the 12 bZIP proteins (AtbZIP36, AtbZIP37, AtbZIP38, OsbZIP23, OsbZIP46, OsbZIP72, WABI5-1, TaABL1, and TabZIP60) played important roles in plant responses to abiotic stress (Choi et al., 2000; Kang et al., 2002; Kim et al., 2004; Finkelstein et al., 2005; Kobayashi et al., 2008; Xiang et al., 2008; Lu et al., 2009; Hossain et al., 2010a; Tang et al., 2012; Xu et al., 2014; Zhang et al., 2015). The phylogenetic analysis and similarity in the conserved motifs indicated that TabZIP111 and TabZIP174 were possibly involved in abiotic stress responses.

### Gene structure analysis

**Figure 3:**
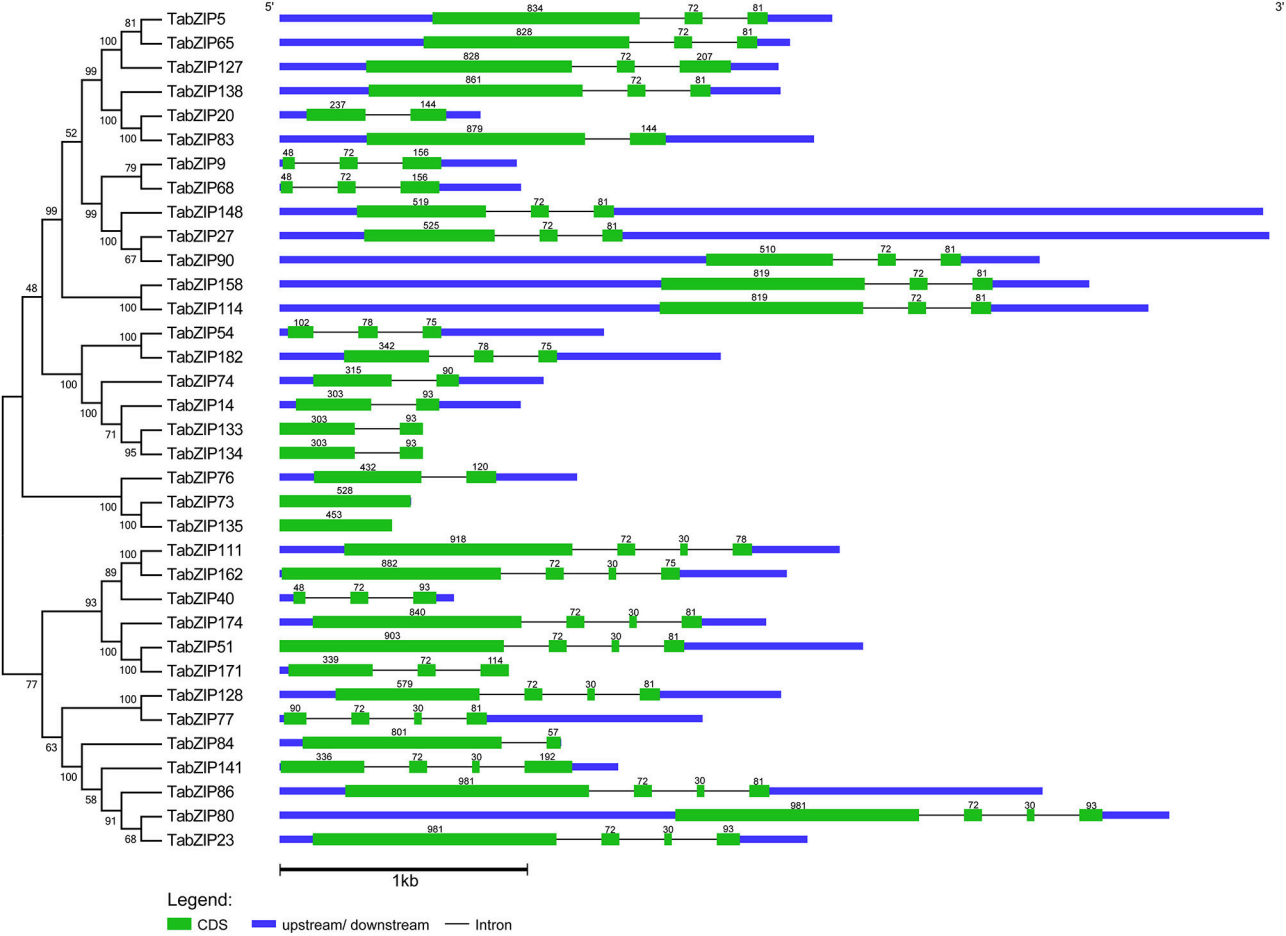
**Phylogenetic analysis (left) and exon–intron structures (right) of 35 novel Subgroup-A ***TabZIP*** genes. (Left)** The phylogenetic analysis was performed using the protein sequences of the 35 novel Subgroup-A *TabZIP* genes. The unrooted phylogenetic tree was generated by the neighbor-joining method using MEGA5.0 software. Numbers above or below branches of the tree indicate bootstrap values. **(Right)** 5′-and 3′-untranslated regions (represented by blue boxes) and exons (represented by green boxes) are drawn to scale. Black lines connecting exons represent introns. Numbers above the exons of each gene structure indicate the size of the exons.

To obtain a deeper insight into the structures of the 35 novel *TabZIP* genes in Subgroup A, we mapped their exon/intron organizations. Only two (5.7%) of these genes were intronless, and the others had 1–3 introns (Figure 3). Specifically, 10 *TabZIP* genes had three introns, 15 had two introns, and 8 had one intron.

Not surprisingly, neighboring *TabZIP* genes in the phylogenetic tree tended to share similar gene structures (Figure 3). *TabZIP9* and *TabZIP68* possessed identical exon/intron organizations, as did *TabZIP23*/*TabZIP80, TabZIP114*/*TabZIP158*, and *TabZIP14*/*TabZIP133*/*TabZIP134*. Except slight variations in the first exon lengths, *TabZIP5* and *TabZIP65*, as well as *TabZIP27, TabZIP90*, and *TabZIP148*, had nearly identical exon/intron compositions. Similarly, *TabZIP80* differed from *TabZIP86* only by 12 nucleotides (nt) in the last exon lengths. Both *TabZIP73* and *TabZIP135* had only one exon, despite a difference in exon length. Differences in exon lengths between these homologous *TabZIP* genes (Table 2) could potentially lead to their functional divergences.

**Table 2:** **Homologous genes within Subgroup A of the wheat bZIP family^a^**.

**Category**	***TabZIP***	**Chromosomal location**
Homologs	*TabZIP5*	1AL
	*TabZIP65*	1BL
	*TabZIP127*	1DL
	*TabZIP9*	2AS
	*TabZIP68*	2BS
	*TabZIP14*	2AL
	*TabZIP74*	2BL
	*TabZIP133*	2DL
	*TabZIP73*	2BL
	*TabZIP135*	2DL
	*TabZIP23/TaABF1*	3AL
	*TabZIP80*	3B
	*TabZIP83*	3B
	*TabZIP138*	3DL
	*TabZIP27*	4AS
	*TabZIP90*	4BL
	*TabZIP148*	4DL
	*WABI5-1*	5A (or 5B)
	*WABI5-2*	5B (or 5A)
	*TabZIP162/WABI5-3*	5DL
	*TabZIP114*	5BL
	*TabZIP158*	5DL
	*TabZIP51/TabZIP60-A*	6AL
	*TabZIP60-B*	6B
	*TabZIP60-D*	6D
	*TabZIP54*	7AL
	*TabZIP182*^b^	7DL
Paralogs (tandem)	*TabZIP133*	2DL
	*TabZIP134*	2DL
	*TabZIP80*	3B
	*TabZIP84*	3B
	*TabZIP86*	3B

a *Homologous genes within the Subgroup A of the wheat bZIP family are divided into two categories (homologs and paralogs). Generally, homologs refer to those homologous genes located at similar positions on three corresponding chromosomes from the A, B, and D sub-genomes of wheat, respectively. Tandem paralogs refer to those homologous genes located at neighboring positions on the same chromosome*.

b *TabZIP54 and TabZIP182 may be homologs*.

In addition, for 30 (85.7%) of these 35 Subgroup-A *TabZIP* genes, the first exon was longer than the subsequent exons in the CDS. Statistical analysis showed that exons of 72, 81, 30, and 93 nt in length appeared frequently within the CDSs of the 35 Subgroup-A *TabZIP* genes, with 23 (72 nt-exon), 13 (81 nt), 10 (30 nt), and 6 (93 nt) occurrences, respectively. Moreover, the exon lengths of the Subgroup-A *TabZIP* genes were all multiples of three without exception (Figure 3), demonstrating that all of their introns were in Phase 0 (P0).

The intron positions within the bZIP domains were also analyzed. Among the 33 intron-containing *TabZIP* genes, 32 had introns within the bZIP domain region. The 35 Subgroup-A *TabZIP* genes were divided into seven patterns (a–g) (Supplementary Figure 2), based on intron numbers and positions, which was consistent with previous studies in rice and maize (Nijhawan et al., 2008; Wei et al., 2012).

A majority (57.1%) of the Subgroup-A *TabZIP* genes were of Pattern a, and these *TabZIP* genes possessed only one intron, which was in P0 and located between the codons encoding the amino acids Gln and Ala (or Ser) in the hinge region (Supplementary Figures 2, 3). This was in accordance with previous studies (Nijhawan et al., 2008; Wei et al., 2012). Patterns b and d were similar in that both had two introns in P0, one within the hinge region and the other within the leucine zipper region. The only difference was that the intron within the leucine zipper region was inserted between Gln at Position +19 and Ala (or Lys) at Position +20 in Pattern b, but was inserted between Glu at Position +21 and Leu at Position +22 in Pattern d. Pattern e had three introns, one in the hinge region and two in the leucine zipper region. Two of them shared the same positions with the two introns of Pattern b, one intervening between the amino acids at Positions −5 and −6 in the hinge region and the other between the amino acids at Positions +19 and +20 in the leucine zipper region. Pattern f had only one intron within the leucine zipper region, and was only found in one *TabZIP* gene. Pattern g lacked any intron in the bZIP domain region and was found in three *TabZIP* genes.

Notably, if present, introns within the hinge region always occurred at the same position, between Gln (or Arg) at Position −6 and Ala (or Ser) at Position −5 (such as Patterns a, b, d, and e), which was in agreement with the findings of a previous study (Nijhawan et al., 2008). However, introns present in the leucine zipper region were at variable positions. In our case, no introns were within the basic region of the bZIP domain. To sum up, *TabZIP* genes with close phylogenetic relationships tended to share similar exon/intron organizations, and the positions of the introns within the hinge regions of the bZIP domains remained well conserved during the evolution of the Subgroup-A *TabZIP* genes.

### Identification of homologous genes

As homologous genes typically retain similar biological roles, identifying homologous genes is important for transferring functional information between genes (Remm et al., 2001). To identify homologous genes within Subgroup A of the wheat bZIP family, pairwise protein sequence alignments were performed using the BLASTP 2.3.0+ program (Altschul et al., 2005). A total of 13 groups of homologous *bZIP* genes were identified from Subgroup A and included two categories: homologs (11 groups) and paralogs (2 groups) (Table 2).

**Figure 4:**
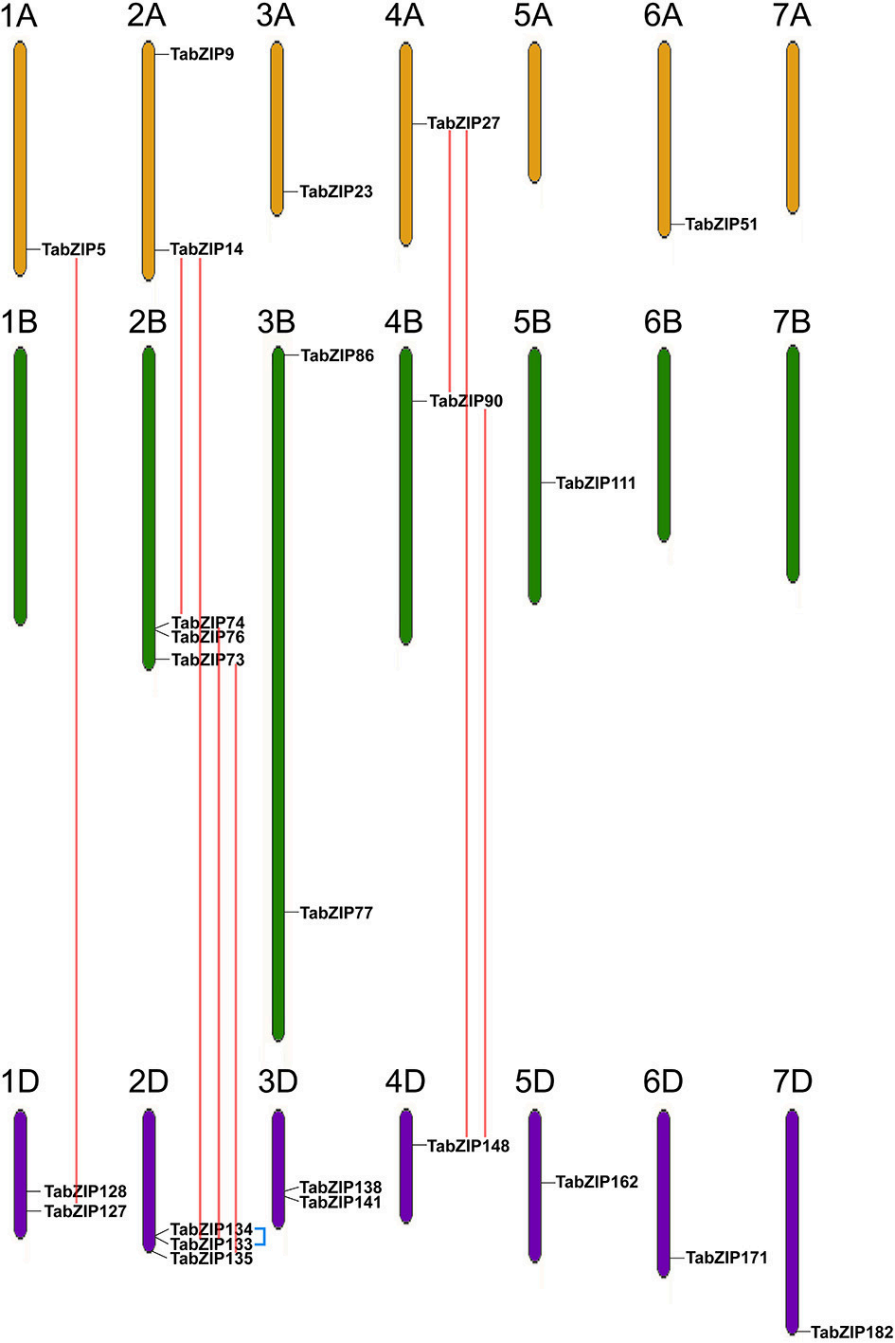
**Chromosomal distribution of Subgroup-A ***TabZIP*** genes**. All wheat chromosomes are drawn to scale based on their actual lengths. 1A–7A, 1B–7B, and 1D–7D represent the seven chromosomes in the A, B, and D sub-genomes of wheat, respectively. Chromosomes from different sub-genomes are indicated by different colors. Only the *TabZIP* genes with available detailed chromosomal location information are shown. Homologs (or paralogs) are linked with red (or blue) straight lines.

Modern wheat (*T. aestivum*, 2*n* = 6*x* = 42, AABBDD) derives from two hybridizations between three gramineous ancestors (Brenchley et al., 2012; Jia et al., 2013). The wheat genome is composed of A, B, and D sub-genomes. In wheat, homologous genes located at similar positions on three corresponding chromosomes from the A, B, and D sub-genomes are generally referred to as homologs. For instance, *TabZIP14, TabZIP74*, and *TabZIP133* were three homologs located at similar positions on the long arms of chromosomes 2A, 2B, and 2D, respectively (Figure 4). They shared high similarities in protein sequences with the aligned region between each pair covering the whole sequences, and their identities were 92, 98, and 94%, respectively.

Tandem paralogs are homologous genes located at neighboring positions of the same chromosome. In this study, we identified two groups of tandem paralogs within Subgroup A of the wheat bZIP family, *TabZIP133*/*TabZIP134* and *TabZIP80*/*TabZIP84*/*TabZIP86* (Table 2). Paralogs *TabZIP133* and *TabZIP134* possessed identical protein sequences and were adjacent to each other in the long arm of chromosome 2D (Figure 4), indicating that this pair of paralogs probably derived from one tandem duplication event, which often led to the generation of paralogs. Paralogs *TabZIP80, TabZIP84*, and *TabZIP86* were all located on chromosome 3B and shared high similarities in protein sequences. Specifically, the aligned region between TabZIP80 (391 aa) and TabZIP86 (387 aa) covered their entire sequences, and the identity within the aligned region was 89%. The aligned region between TabZIP80 and TabZIP84 (286 aa) covered the entire TabZIP84 sequence and 73% of the TabZIP80 sequence, with an identity of 90%. The aligned region between TabZIP84 and TabZIP86 covered the entire TabZIP84 sequence and 74% of the TabZIP86 sequence, with an identity of 87%. This suggested that this paralogous group (*TabZIP80*/*TabZIP84*/*TabZIP86*) might have been derived from two tandem duplication events. Further genomic sequence alignment among them showed that the unspliced transcript sequence of *TabZIP84* (including 5′- and 3′-untranslated regions, two exons and one intron) was aligned with only a portion of the unspliced transcript sequence of *TabZIP80* (or *TabZIP86*) (data not shown), indicating that the gene *TabZIP84* derived from only a segment of *TabZIP80* (or *TabZIP86*).

### Expression profiles of subgroup-A *TabZIP* genes in response to various abiotic stresses

**Figure 5:**
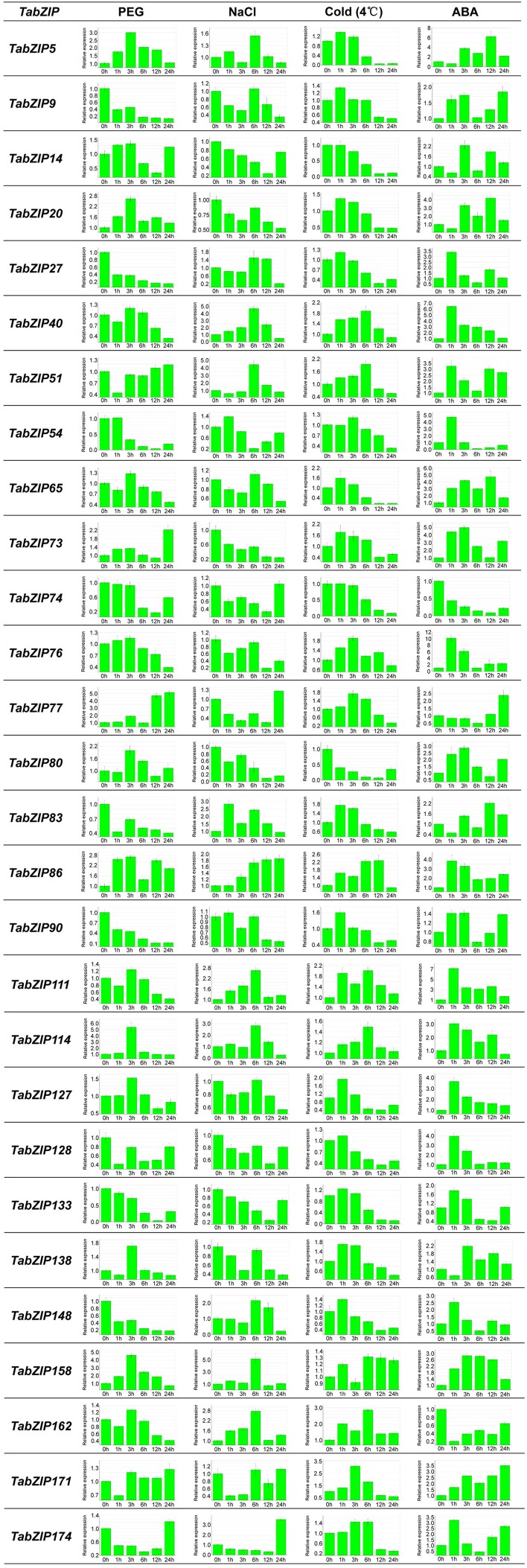
**Expression profiles of Subgroup-A ***TabZIP*** genes in response to multiple abiotic stress and ABA treatments**. The 2^−ΔΔC_T_^ method was used to calculate the relative expression levels of *TabZIP* genes. The expression of each *TabZIP* gene at 0 h after treatment is regarded as a reference, and other values represent the expression levels relative to the reference. Mean values and SDs were obtained from three replicates.

Compelling evidence demonstrated that the expression of Subgroup-A *bZIP* genes in *Arabidopsis* and rice was induced by ABA and various abiotic stress treatments (Choi et al., 2000; Xiang et al., 2008; Hossain et al., 2010b; Tang et al., 2012). To further confirm whether the expression of Subgroup-A *bZIP* genes in wheat was induced by different abiotic stresses, quantitative real-time PCR (qRT-PCR) was performed to analyze the expression profiles of these *TabZIP* genes following PEG, NaCl, cold (4°C) or ABA treatments. Overall, the expression of 28 of these 35 Subgroup-A *TabZIP* genes responded to all four treatments and exhibited complicated variation trends following the treatments (Figure 5).

Among the expression profiles of the 28 *TabZIP* genes under the four different treatments (28 × 4 = 112), 20 exhibited a variation trend in which the transcript level gradually ascended and reached a maximum, followed by a continuous decline. These included *TabZIP5* (under PEG treatment), *TabZIP9* (cold), *TabZIP20* (cold), *TabZIP40* (NaCl, cold or ABA), *TabZIP51* (cold), *TabZIP65* (cold), *TabZIP76* (PEG), *TabZIP77* (cold), *TabZIP83* (cold), *TabZIP114* (PEG or cold), *TabZIP127* (ABA), *TabZIP133* (cold), *TabZIP138* (cold), *TabZIP158* (PEG or ABA), *TabZIP171* (cold), and *TabZIP174* (cold).

In addition, 10 other expression profiles had an inverse variation trend in which the transcripts decreased to the lowest level and then increased. These included *TabZIP14* (NaCl or cold), *TabZIP74* (PEG or ABA), *TabZIP77* (ABA), *TabZIP80* (cold), *TabZIP133* (PEG or NaCl), and *TabZIP174* (PEG or NaCl).

Under the NaCl treatment, the expression level of *TabZIP86* continuously increased and reached its maximum at 24 h after treatment. In contrast, for *TabZIP27* (PEG) and *TabZIP74* (cold), the transcript levels declined continuously until reaching their lowest levels at 24 h after treatment. After the 24-h PEG treatment, the expression level of *TabZIP27* was only one-seventh of that before treatment. The transcript level of *TabZIP74* after the 24-h cold treatment dropped to one-tenth of that before treatment. Thus, the expression levels of *TabZIP27* and *TabZIP74* were down-regulated by PEG and cold treatments, respectively.

Notably, the expression of several *TabZIP* genes (such as *TabZIP40, TabZIP76*, and *TabZIP111*) responded rapidly to the ABA treatment because their expression levels increased rapidly immediately after a 1-h exposure to exogenous ABA. For instance, the transcript level of *TabZIP76* was more than 10 times that before treatment, while the transcript levels of *TabZIP40* and *TabZIP111* were ~6.5 and 7 times those before treatment, respectively.

Interestingly, some homologs (or paralogs) exhibited similar expression profiles following certain treatments, such as *TabZIP5*/*TabZIP65* (cold), *TabZIP14*/*TabZIP133* (NaCl), *TabZIP74*/*TabZIP133* (PEG), *TabZIP27*/*TabZIP90* (PEG), *TabZIP27*/*TabZIP90*/*TabZIP148* (cold), *TabZIP27*/*TabZIP148* (NaCl or ABA), *TabZIP83*/*TabZIP138* (cold), and *TabZIP114*/*TabZIP158* (PEG). However, most homologous *TabZIP* genes were expressed differently under the same treatments (Figure 5). Indeed, *TabZIP80* and *TabZIP86* even displayed inverse variation trends of expression under the NaCl (or cold) treatment. After the NaCl treatment, *TabZIP86* transcripts accumulated gradually and reached a maximum at 24 h post-treatment, while, in contrast, *TabZIP80* expression was down-regulated by NaCl, with its transcript level dropping to a relatively lower level after the 24-h treatment. This suggested that homologous *TabZIP* genes in Subgroup A had undergone expression pattern shifts.

### Subcellular localization of subgroup-A TabZIP proteins in wheat protoplasts

Our phylogenetic and conserved motif analyses suggested that TabZIP174 (belonging to Clade A1) were more likely involved in abiotic stress response. In addition to TabZIP174, several Subgroup-A TabZIP members (TabZIP9, TabZIP27, TabZIP74, and TabZIP138) were selected from other clades to confirm their subcellular localization. First, the NLSs were predicted using their protein sequences. A NLS, consisting of 4–8 amino acid (e.g., Pro, Lys, or Arg) residues, commonly exists within a transcription factor protein (Kalderon et al., 1984). The basic region within the bZIP domain generally includes a NLS followed by an invariant motif (N-X_7_-R/K) that is responsible for contacting the DNA (Jakoby et al., 2002). According to the NLS prediction result by NucPred (Brameier et al., 2007), TabZIP27 possessed a NLS sequence (RRKKR) positioned three amino acid residues ahead of the N-terminus of the N-X_7_-R/K motif, and TabZIP138 also had a NLS sequence (GRRKR) located away from the N-terminus of the bZIP domain.

To further examine the subcellular localizations of these five TabZIP proteins, wheat protoplasts were separated from mesophyll tissue and then transformed with *35S::TabZIP9-GFP, 35S::TabZIP27-GFP, 35S::TabZIP74-GFP, 35S::TabZIP138-GFP*, and *35S::TabZIP174-GFP* fusion vectors, respectively. The *35S::GFP* vector served as the control. Subsequently, GFP expression was monitored by confocal microscopy at 18 h after PEG transformation.

**Figure 6:**
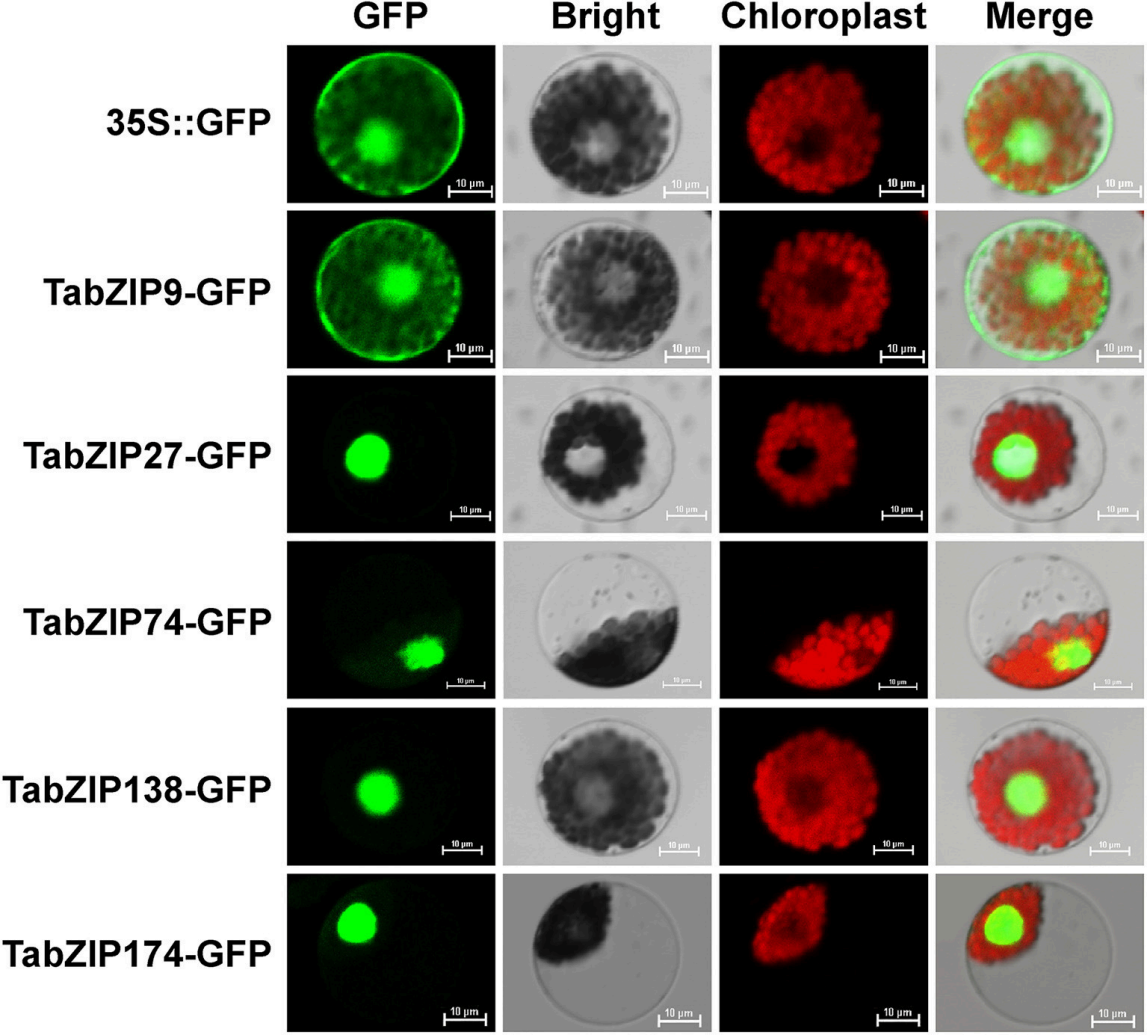
**Subcellular localization of TabZIP-GFP fusion proteins in wheat mesophyll protoplasts**. The *35S::TabZIP9-GFP, 35S::TabZIP27-GFP, 35S::TabZIP74-GFP, 35S::TabZIP138-GFP*, and *35S::TabZIP174-GFP* fusion vectors, and *35S::GFP* control vector were independently transformed into wheat mesophyll protoplasts by PEG transfection. Green fluorescent was monitored under a laser scanning confocal microscope.

Green fluorescence was detected in the nucleus of wheat protoplasts for TabZIP27-GFP, TabZIP74-GFP, TabZIP138-GFP, and TabZIP174-GFP fusion proteins (Figure 6), indicating that TabZIP27, TabZIP74, TabZIP138, and TabZIP174 proteins were exclusively localized in the nucleus. Nevertheless, green fluorescence of TabZIP9-GFP fusion protein was simultaneously present in the nucleus, cytoplasm, and cell membrane.

### Germination rate and primary root growth of *TabZIP174* transgenic *Arabidopsis*

The foregoing phylogenetic and conserved motif analyses suggested that TabZIP174 potentially participated in regulating abiotic stress response. To further investigate the role of *TabZIP174* in response to abiotic stress, *35S::TabZIP174* transgenic *Arabidopsis* lines were generated. To examine differences in the germination rates and primary root growth between *TabZIP174* transgenic and WT *Arabidopsis*, seeds of three transgenic lines (OE-2, OE-4, and OE-5) and WT were germinated on MS medium.

**Figure 7:**
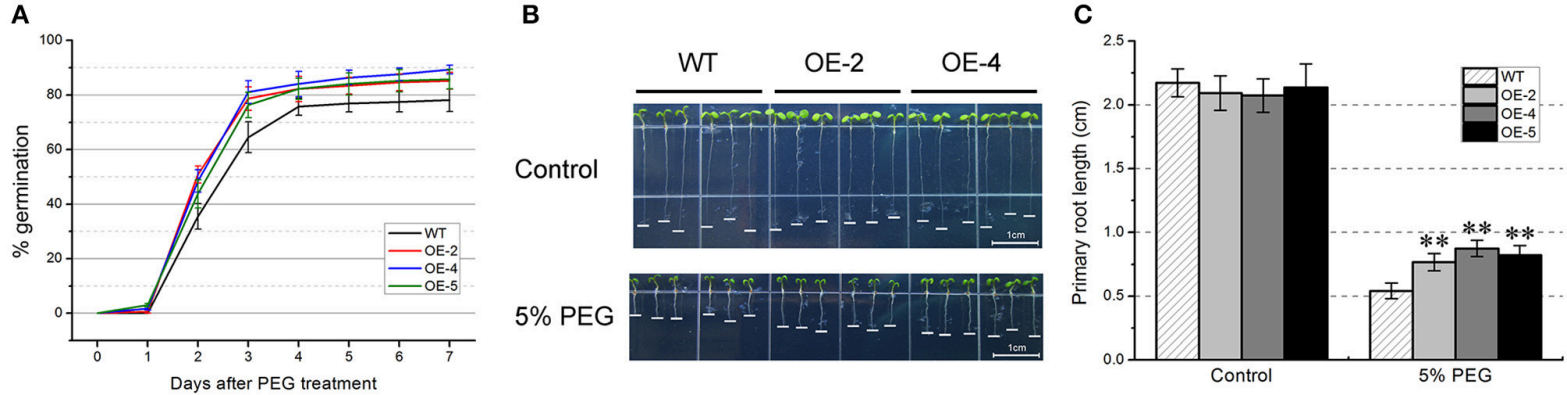
**Comparison of germination rates and primary root lengths between ***TabZIP174*** transgenic and wild type (WT) ***Arabidopsis*****. OE-2, OE-4 and OE-5 represent three *TabZIP174* transgenic *Arabidopsis* lines. **(A)** Germination rates of *TabZIP174* transgenic and WT seeds after the 5% (w/v) PEG treatment. Germination rates were determined daily for 7 days following 2-day stratification. In each of three independent repetitions, 169 (13 × 13) seeds per *Arabidopsis* line were used. Data represent the means ± SDs. **(B)** Primary root growth of *TabZIP174* transgenic and WT plants on Murashige and Skoog (MS) medium with or without 5% PEG. Thirty plants per *Arabidopsis* line were used in each of three independent repetitions. Representative transgenic and WT plants were photographed after the 7-day growth. **(C)** Statistical analysis of primary root lengths. Primary root lengths were measured after the 7-day growth. Data represent the means ± SDs. Asterisks indicate statistically significant differences between transgenic and WT plants (Student's *t*-test, ^**^P < 0.01).

Under normal conditions, there were no significant differences in germination rates and primary root lengths between the transgenic lines (OE-2, OE-4, and OE-5) and WT. However, 3 days after the PEG treatment, the germination rates of the transgenic lines (OE-2, OE-4, and OE-5) were higher than that of WT (Figure 7A), and the differences between OE-2 and WT and between OE-4 and WT both reached the significant level (*t*-test, *P* < 0.05). Compared with WT, the germination rates of the transgenic lines OE-2, OE-4, and OE-5 increased by ~14, 17, and 12%, respectively.

In the presence of PEG, primary root elongation was significantly inhibited for both the transgenic lines and WT, however, the primary root lengths of lines OE-2, OE-4, and OE-5 were significantly greater than those of WT plants (*t*-test, *P* < 0.01) (Figures 7B,C). This indicated that the inhibitory effect of PEG on primary root growth was more serious in WT than in the transgenic plants. Thus, our results suggested that *TabZIP174* overexpression enhanced the tolerance of transgenic *Arabidopsis* to the imposed drought stress.

### *TabZIP174*-overexpressing transgenic *Arabidopsis* had enhanced drought tolerance

Under well-watered conditions, there were no evident morphological differences between transgenic and WT *Arabidopsis* throughout their life cycles.

**Figure 8:**
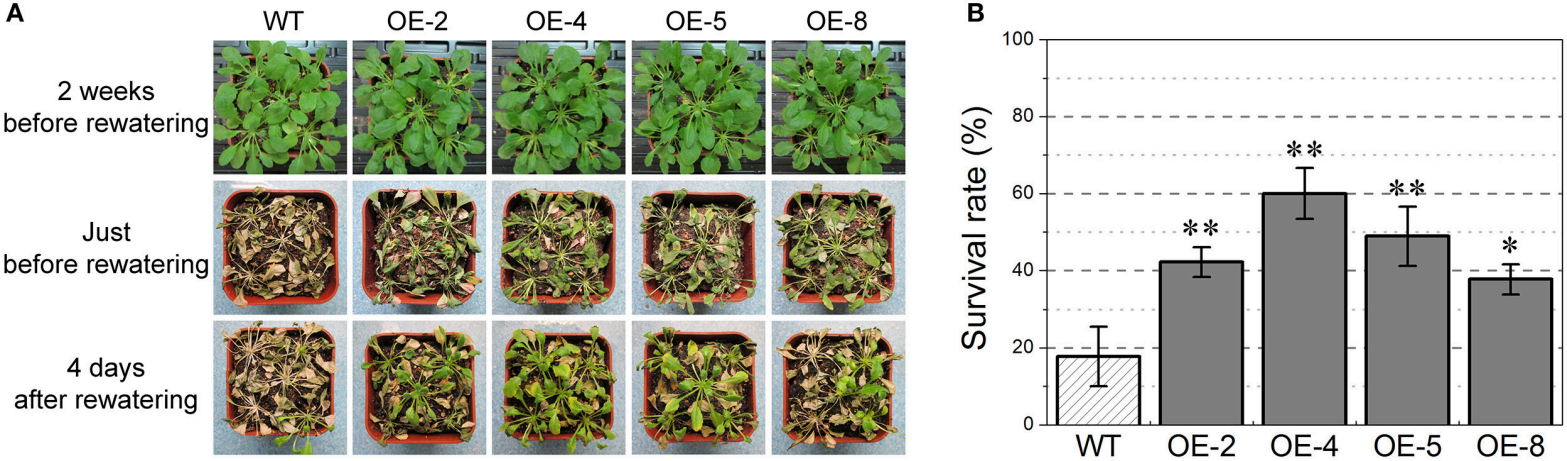
**Overexpression of ***TabZIP174*** in transgenic ***Arabidopsis*** improved drought tolerance**. Fifteen plants per *Arabidopsis* line were used in each of three independent repetitions. OE-2, OE-4, OE-5, and OE-8 represent four *TabZIP174* transgenic *Arabidopsis* lines. **(A)** Phenotypes of *TabZIP174* transgenic and wild type (WT) plants under water deficit conditions and after rewatering. **(B)** Survival rates of *TabZIP174* transgenic and WT plants 4 days after rewatering. Data represent the means ± SDs. Asterisks indicate statistically significant differences between transgenic and WT plants (Student's *t*-test, ^*^P < 0.05, ^**^P < 0.01).

Four *TabZIP174* transgenic lines were selected for testing in soil to characterize their performances under drought stress. At the early stage of the drought treatment (e.g., 2 weeks before rewatering), *TabZIP174* transgenic and WT plants grew normally, with no notable phenotypic differences between them (Figure 8A: the upper row).

Eighteen days after withholding water, the lower rosette leaves of WT plants showed slight wilting, whereas *TabZIP174* plants still grew normally. After 4-week drought treatment (just before rewatering), most WT plants were severely wilted and a number of rosette leaves were yellow or dead. In contrast, although most *TabZIP174* plants were wilted and many rosette leaves were severely curled, most leaves remained green and only some *TabZIP174* plants displayed symptoms of severe water deficit (Figure 8A: the middle row).

Four days after rewatering, 38–60% of the *TabZIP174* plants had survived, whereas only 18% of the WT plants had survived (Figure 8A: the lower row, Figure 8B). The survival rates of lines OE-2, OE-4, and OE-5 were 42, 60, and 49%, respectively, which were much higher (*t*-test, *P* < 0.01) than that of WT (18%). Thus, the overexpression of *TabZIP174* greatly improved drought resistance in transgenic *Arabidopsis*.

### Physiological changes in *TabZIP174* transgenic *Arabidopsis*

**Figure 9:**
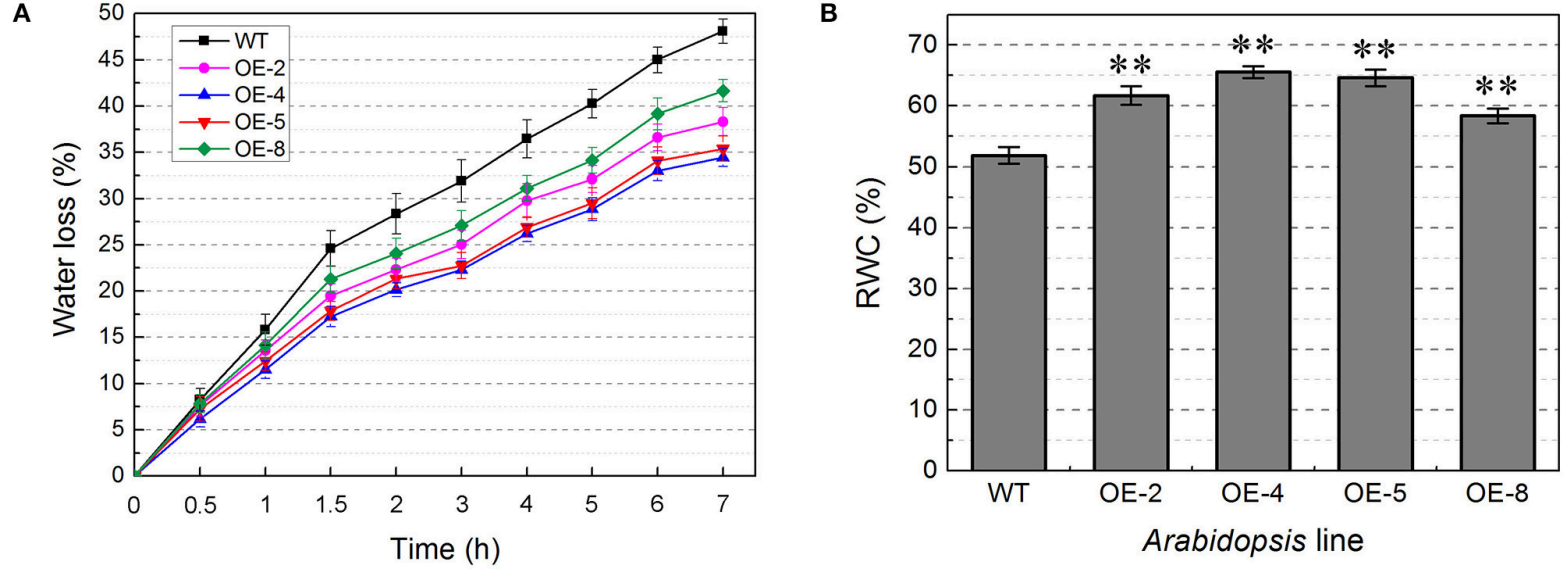
*****TabZIP174*** transgenic plants have stronger water retention ability**. OE-2, OE-4, OE-5, and OE-8 represent four *TabZIP174* transgenic lines. Data represent the means ± SDs. **(A)** Comparison of water loss rates for detached rosettes between transgenic and wild type (WT) plants under dehydration conditions. **(B)** Comparison of relative water contents (RWCs) for detached rosettes between transgenic and WT plants after a 7-h dehydration treatment. Asterisks indicate statistically significant differences between transgenic and WT plants (Student's *t*-test, ^**^P < 0.01).

Water loss rate is an important index that has been used to evaluate the water status of plants under water deficit conditions (Mao et al., 2012). In the present study, the water loss rates of the detached rosettes for transgenic lines were lower than that of WT plants, and the final relative water contents of transgenic lines were significantly higher than that of WT (*t*-test, *P* < 0.01) (Figure 9), indicating that *TabZIP174* transgenic plants had stronger water retention capacity.

**Figure 10:**
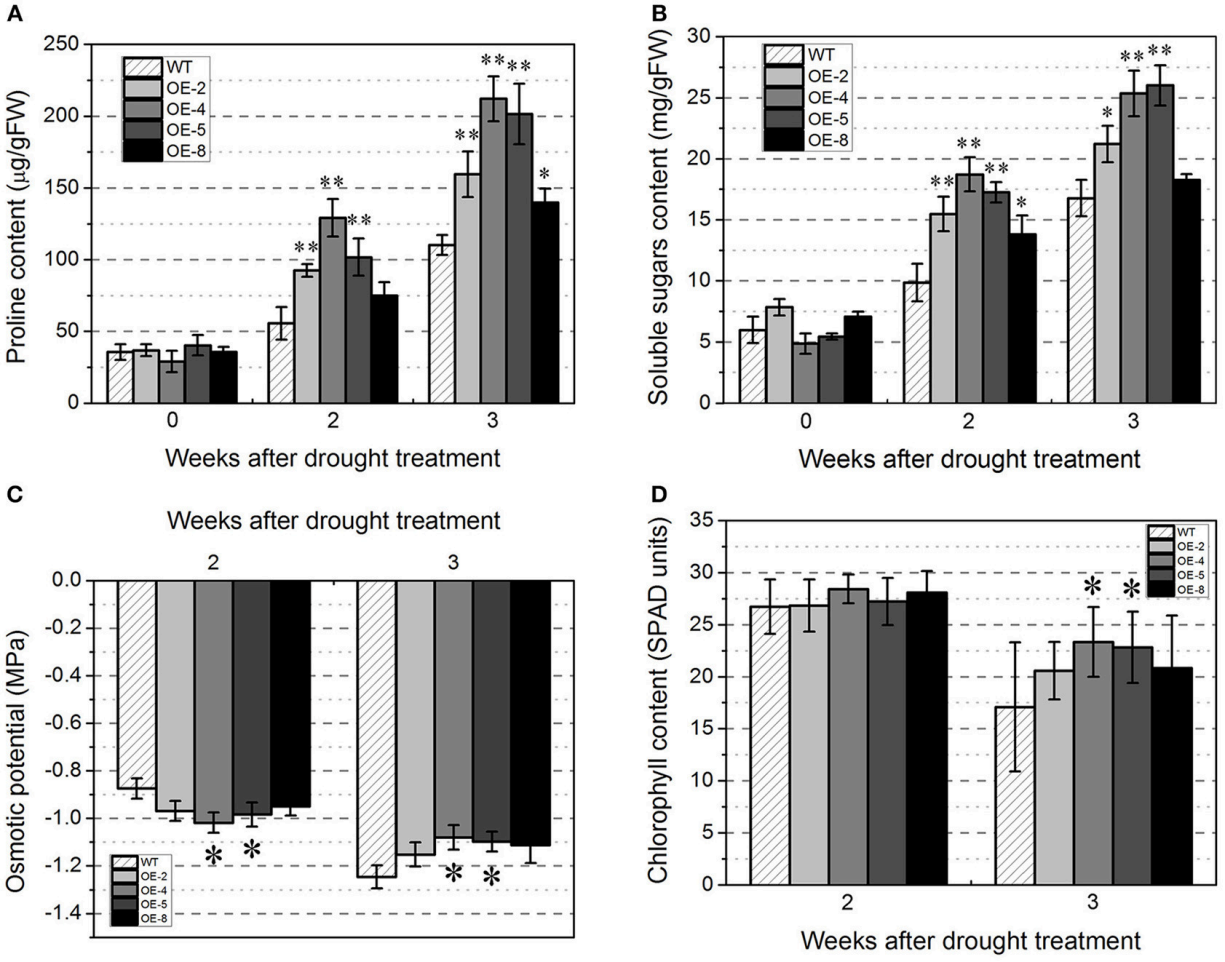
**Physiological characterization of ***TabZIP174*** transgenic and wild type (WT) ***Arabidopsis*** under water deficit conditions**. OE-2, OE-4, OE-5, and OE-8 represent four *TabZIP174* transgenic lines. “0,” “2,” and “3” under the horizontal axis refer to three time-points, before drought treatment, 2-weeks after drought treatment, and 3-weeks after drought treatment, respectively. Data represent the means ± SDs. Asterisks indicate statistically significant differences between transgenic and WT plants (Student's *t*-test, ^*^P < 0.05, ^**^P < 0.01). **(A)** Free proline content. **(B)** Total soluble sugars content. **(C)** Osmotic potential. **(D)** Chlorophyll content.

To explore whether *TabZIP174* overexpression influenced proline accumulation, the free proline contents in the transgenic and WT plants were measured. After drought treatment, the proline contents of the transgenic lines were higher than those of the WT plants. And the difference between each transgenic line (OE-2, OE-4, or OE-5) and WT reached the significant level (*t*-test, *P* < 0.01) whether 2 or 3 weeks after drought treatment (Figure 10A).

The contents of total soluble sugars were also measured in the transgenic and WT plants under drought stress to determine whether the enhanced drought tolerance of the transgenic plants was associated with soluble sugars. Under water deficit conditions, the soluble sugar contents obviously increased compared with those before drought treatment for both transgenic and WT plants, and notably, the soluble sugar contents of transgenic lines OE-2, OE-4, and OE-5 were significantly higher than that of WT 2 weeks after drought treatment (*t*-test, *P* < 0.01) (Figure 10B). The results suggested that *TabZIP174* probably participated in proline and carbohydrate metabolism.

To retain a relatively stable intracellular environment under abiotic stress conditions, many plants decrease their intracellular osmotic potential by accumulating organic osmolytes (such as proline, mannitol, and glycine betaine) in cells (Zhang et al., 2011). Our result revealed that 2 weeks after drought treatment, the transgenic plants had lower osmotic potential than WT. In contrast, the osmotic potential was higher in the transgenic plants than in WT 3 weeks after drought treatment (Figure 10C). And whether 2 or 3 weeks after drought treatment, the difference between transgenic line OE-4 (or OE-5) and WT reached the significant level (*t*-test, *P* < 0.05). Compared with WT plants, *TabZIP174* transgenic lines maintained a relatively stable osmotic potential under water deficit conditions.

Additionally, the leaf chlorophyll content was measured to further determine possible physiological differences between transgenic and WT plants. Two weeks after drought treatment, the rosette leaves of all of the transgenic and WT plants remained green (Figure 8A: the upper row); with no evident difference in the chlorophyll content (Figure 10D). Three weeks after drought treatment, however, transgenic lines OE-4 and OE-5 had significantly higher chlorophyll contents than WT (*t*-test, *P* < 0.05) (Figure 10D). The severe drought resulted in a more rapid decrease in the chlorophyll content of WT than transgenic plants. This suggested that the overexpression of *TabZIP174* could directly or indirectly slow chlorophyll degradation in leaves of transgenic plants under severe drought conditions.

### Enhanced expression of stress-responsive genes in *TabZIP174* transgenic plants

**Figure 11:**
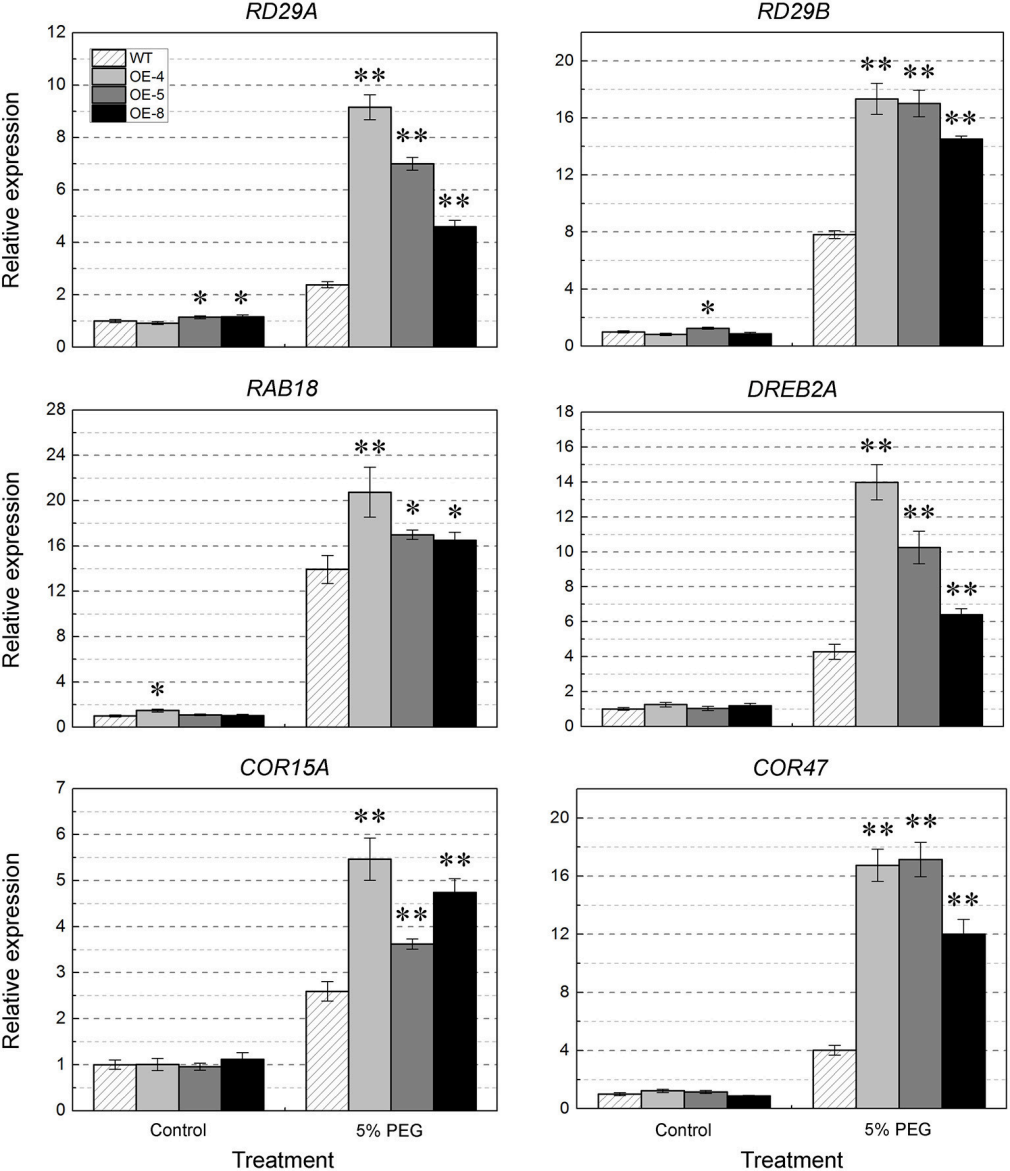
**Expression of stress-responsive genes in ***TabZIP174*** transgenic and wild type (WT) ***Arabidopsis*** treated with PEG**. OE-4, OE-5, and OE-9 represent three *TabZIP174* transgenic lines. Total RNA was obtained from two-week-old seedlings grown under normal conditions or 5% (w/v) PEG treatment for 2 h. Transcript levels were measured using qRT-PCR of *RD29A, RD29B, RAB18, DREB2A, COR15A*, and *COR47* under normal conditions and PEG treatment. *Arabidopsis Actin2* was used as the internal control. Data represent the means ± SDs. Asterisks indicate statistically significant differences between transgenic and WT plants (Student's *t*-test, ^*^P < 0.05, ^**^P < 0.01).

Our results of phenotypic studies showed that *TabZIP174* transgenic lines had improved resistance to drought stress. To further explore the underlying molecular mechanisms, we performed the expression analyses of nine stress-response-related genes in transgenic and WT plants under normal and water deficit conditions. Transcript levels of six genes (*RD29A, RD29B, RAB18, DREB2A, COR15A*, and *COR47*) were similar between transgenic lines and WT under normal conditions, whereas after PEG treatment, their transcript levels were significantly higher in transgenic lines than in WT (*t*-test, *P* < 0.05 or *P* < 0.01) (Figure 11). However, there were no significant differences in transcript levels of the other three genes (*P5CS, ABI1*, and *ABI2*) between transgenic lines and WT (data not shown).

## Discussion

In this study, we performed a series of bioinformatic analyses of the Subgroup-A members of the wheat bZIP transcription factor family for the first time. Among the Subgroup-A bZIP proteins, 15 bZIPs (7 TabZIPs, 3 AtbZIPs, and 5 OsbZIPs) played important roles in regulating abiotic stress responses of plants (Kang et al., 2002; Kim et al., 2004; Kobayashi et al., 2008; Xiang et al., 2008; Zou et al., 2008; Lu et al., 2009; Hossain et al., 2010b; Tang et al., 2012; Xu et al., 2014; Zhang et al., 2015). Most (13/15) of these 15 bZIPs clustered in Clade A1 of the phylogenetic tree (Figure 1). Thus, the study of Subgroup-A *bZIP* genes, especially those in Clade A1, should be emphasized because of their potential importance in responses to abiotic stress. In addition, the expression of most *TabZIP* genes in Subgroup A were induced by PEG, NaCl, cold, and exogenous ABA treatments, implying that they might play roles in response to ABA or various abiotic stresses, just as those *bZIP* genes in *Arabidopsis* and rice (Choi et al., 2000; Xiang et al., 2008; Hossain et al., 2010b; Tang et al., 2012). The complicated variation trends in the gene expression reflected the complexity of the possible regulatory mechanisms of abiotic stress responses by transcription factors. The subcellular localization results confirmed that most TabZIP proteins acted as transcription factors and performed their functions in the nucleus (Figure 6). Our analyses proposed the importance of the wheat Subgroup-A *bZIP* genes in abiotic stress responses, and laid a foundation for further studies on these *TabZIP* genes, which would facilitate the excavation of valuable gene resources with great potential for genetic improvement of abiotic stress tolerance in crops.

A novel Subgroup-A *bZIP* gene, *TabZIP174*, was cloned from wheat in this study. *TabZIP174* was predominantly expressed in leaf (Supplementary Figure 4) and encoded a putative protein of 340 aa, with a theoretical isoelectric point of 8.84 and a molecular weight of 36.78 kDa (ExPASy, Compute pI/Mw tool, http://web.expasy.org/compute_pi/). TabZIP174 protein possessed a typical bZIP conserved domain and was exclusively localized in the nucleus (Figure 6), which was consistent with its putative function as a transcription factor. Sequence analysis showed that TabZIP174 had a highly similar protein sequence and shared the vast majority of conserved motifs with OsbZIP23 and OsbZIP46 (Figure 1, Supplementary Figure 1), suggesting that they might have similar roles in regulating abiotic stress responses (Xiang et al., 2008; Hossain et al., 2010a; Tang et al., 2012).

To investigate the role of *TabZIP174* in abiotic stress response, *TabZIP174* was transformed into *Arabidopsis*, and its overexpression was confirmed by reverse transcription PCR. Germination, primary root growth and drought tolerance assays demonstrated that the overexpression of *TabZIP174* in transgenic *Arabidopsis* significantly enhanced drought tolerance (Figures 7, 8). Our results were consistent with previous studies on other Subgroup-A bZIP proteins. For instance, the overexpression of *TaABL1* in transgenic plants conferred enhanced resistance to drought stress (Xu et al., 2014). In addition, our expression analysis demonstrated that the expression of *TabZIP174* was rapidly responsive to both PEG and ABA treatments (Figure 5). Our findings suggested that *TabZIP174* participated in regulating plant response to drought stress, likely through an ABA-dependent pathway, which was similar to the regulatory mechanisms of several other bZIP transcription factors in Subgroup A (Kang et al., 2002; Xiang et al., 2008).

Interestingly, *TabZIP174* transgenic plants exhibited higher germination rates and longer primary roots than WT plants after the PEG treatment (Figures 7A–C), suggesting that the overexpression of *TabZIP174* weakened the adverse effects of PEG on germination and primary root growth. However, there were no evident differences in both germination rates (data not shown) and primary root lengths (Figures 7B,C) between transgenic and WT plants under normal conditions. Although *TabZIP174* was constitutively overexpressed in transgenic plants under the control of CaMV 35S promoter, TabZIP174 protein may not be activated under normal conditions. Besides, under normal conditions, the expression levels of six stress-responsive genes were similar between transgenic lines and WT (Figure 11), which indicated that the overexpression of *TabZIP174* was not sufficient to activate transcription of downstream target genes. As reported previously, ABA-activated SnRK2 protein kinases regulated ABA-induced genes by phosphorylating AREBs, such as TRAB1 (Kobayashi et al., 2005). The potential phosphorylation sites of TabZIP174 included 5 Ser, 10 Thr, and 1 Tyr residues (NetPhos 2.0 Server, http://www.cbs.dtu.dk/services/NetPhos/). The activation of TabZIP174 might require its own phosphorylation by ABA-activated SnRK2 protein kinases (Kobayashi et al., 2005; Furihata et al., 2006; Fujita et al., 2013), given that the PEG treatment could cause an increase in the endogenous ABA level (Lata and Prasad, 2011; Fujita et al., 2013; Chiappetta et al., 2015).

Gene overexpression might cause the growth retardation of transgenic plants (Kasuga et al., 1999; Kim et al., 2004; Maruyama et al., 2004; Dai et al., 2007), restricting the applicability of target genes in transgenic breeding. The morphological features of *TabZIP174* transgenic plants were closely monitored, and no obvious adverse effects were observed, indicating the potential of *TabZIP174* in plant breeding.

Adverse environmental factors often cause physiological changes in plants. Meanwhile, physiological indices can be used to evaluate the abiotic stress resistance of crops (Mao et al., 2012). Osmotic adjustment is defined as the decrease in osmotic potential achieved by osmolyte accumulation in response to osmotic stress, and is regarded as a beneficial mechanism for drought adaptation in some crop species (Girma and Krieg, 1992; Subbarao et al., 2000; Chen and Jiang, 2010). Generally, a higher capacity in the osmotic adjustment indicates a stronger tolerance and adaptation to osmotic stress. Osmotic potential is an effective physiological index, which is used to directly reflect the osmotic adjustment capability and to evaluate the osmotic stress resistance of plants (Mao et al., 2012). In this study, our result of osmotic potential measurement indicated that *TabZIP174* overexpression enhanced the osmotic adjustment capacity of transgenic *Arabidopsis* (Figure 10C). Proline or soluble sugars accumulation is a common physiological response to water stress in plants. Proline and soluble sugars are both regarded as important osmolytes in osmotic adjustment and play vital roles in weakening the adverse effects of osmotic stress (Kameli and Lösel, 1993, 1995, 1996; Igarashi et al., 1997; Kumar et al., 2003; Bartels and Sunkar, 2005; Claussen, 2005; Verbruggen and Hermans, 2008; Zhang et al., 2011; Mao et al., 2012). In this study, our results suggested that *TabZIP174* overexpression accelerated the accumulation of proline and soluble sugars (Figures 10A,B), and that their rapid accumulation in transgenic plants probably contributed to the enhanced tolerance to drought stress. However, the osmotic potential for four transgenic lines was not completely consistent with either their proline or soluble sugar contents, implying that proline and soluble sugars might only partially account for the osmotic potential changes. At the early stage of drought treatment (e.g., 2 weeks after withholding water), transgenic plants possessed relatively lower osmotic potential, which indicated higher water retention capability. Therefore, at the later stage of drought treatment (e.g., 2 weeks after withholding water), transgenic plants had higher osmotic potential because of higher water contents.

Previous studies showed that the overexpression of maize *ZmbZIP72* significantly increased the transcript levels of *RD29B, RAB18*, and *HIS1-3* and conferred enhanced abiotic stress tolerance in transgenic *Arabidopsis* (Ying et al., 2012). *TaABL1* activated the expression of two stress-responsive genes (*RD29B* and *RAB18*) and two guard cell ion channel genes (*KAT1* and *KAT2*) (Xu et al., 2014). *TabZIP60* functioned in response to multiple abiotic stresses via an ABA-dependent pathway and up-regulated the expression of *RD29A, RD29B, MYB2, COR47, RD20, DREB2A*, and *ERD6* under salt stress conditions (Zhang et al., 2015). Higher expressions of six stress-responsive genes (*RD29A, RD29B, RAB18, DREB2A, COR15A*, and *COR47*) were detected in *TabZIP174* transgenic lines than in WT under water deficit conditions (Figure 11). *RD29A* possesses an ABA-responsive element (ABRE) and a dehydration-responsive element (DRE) in its promoter region, and is induced by drought, cold, and ABA treatments (Shinozaki and Yamaguchi-Shinozaki, 1997). The significant increase in transcription levels of *RD29A, RD29B, RAB18*, and *COR15A* (encoding low-molecular-weight hydrophilic proteins) accelerates osmolyte accumulation in cells, leading to the decrease of osmotic potential, and reduction of water loss rates under drought stress conditions (Lång and Palva, 1992; Yamaguchi-Shinozaki and Shinozaki, 1993; Zhou et al., 2009; Mao et al., 2012).

In conclusion, both phenotypic and physiological evidence demonstrated that the overexpression of *TabZIP174* conferred the enhanced drought tolerance in transgenic *Arabidopsis*. In our view, the improved drought resistance was likely due to the increased osmotic adjustment capacity. The increased osmotic adjustment capacity enabled plants to maintain relatively lower cellular osmotic potential at the early stage of water deficit, which facilitated water retention and decreased water loss, thus strengthening drought resistance.

## Author contributions

XL conceived of the study, performed the bioinformatics analysis and the experimental work, and wrote the manuscript. SG and YT helped in conceiving of the study and revised the manuscript. BF, FZ, and LZ helped in experimental work and data analysis. LM and CZ contributed with valuable discussion. All authors read and approved the final manuscript.

## Funding

This work was financially supported by National Science and Technology Support Program (2013BAD04B02), 948 Projects of the Ministry of Agriculture (2015-Z39, 2016-X58), National Natural Science Foundation of China (31571641), Beijing Municipal Science and Technology Plan Project (Z141100002314018), and Beijing Natural Science Foundation (6162009).

### Conflict of interest statement

The authors declare that the research was conducted in the absence of any commercial or financial relationships that could be construed as a potential conflict of interest.

## Supplementary material

### Availability of data and material

The datasets supporting the conclusions of this article are included within the article and its Supplementary Material.

## Abbreviations

bZIP: Basic leucine zipper
ABA: Abscisic acid
ABF: ABRE binding factor
AREB: ABA response element binding factor
DPBF: *Dc3* promoter binding factor
TriFLDB: Triticeae full-length CDS database
MEME: Multiple expectation maximization for motif elicitation
GSDS: Gene structure display server
nt: Nucleotide
aa: Amino acid
NLS: Nuclear localization signal
CaMV 35S: Cauliflower mosaic virus 35S
GFP: Green fluorescent protein
PEG: Polyethylene glycol
PCR: Polymerase chain reaction
qRT-PCR: Quantitative real-time polymerase chain reaction
WT: Wild type
OE: Overexpression
MS: Murashige and Skoog.
