# Supplementary figures and images for: Bioinformatic Analyses of Subgroup-A Members of the Wheat bZIP Transcription Factor Family and Functional Identification of TabZIP174 Involved in Drought Stress Response
Source: Front Plant Sci. 2016 Nov 16;7:1643. doi: 10.3389/fpls.2016.01643 (PMC5110565; doi:10.3389/fpls.2016.01643)

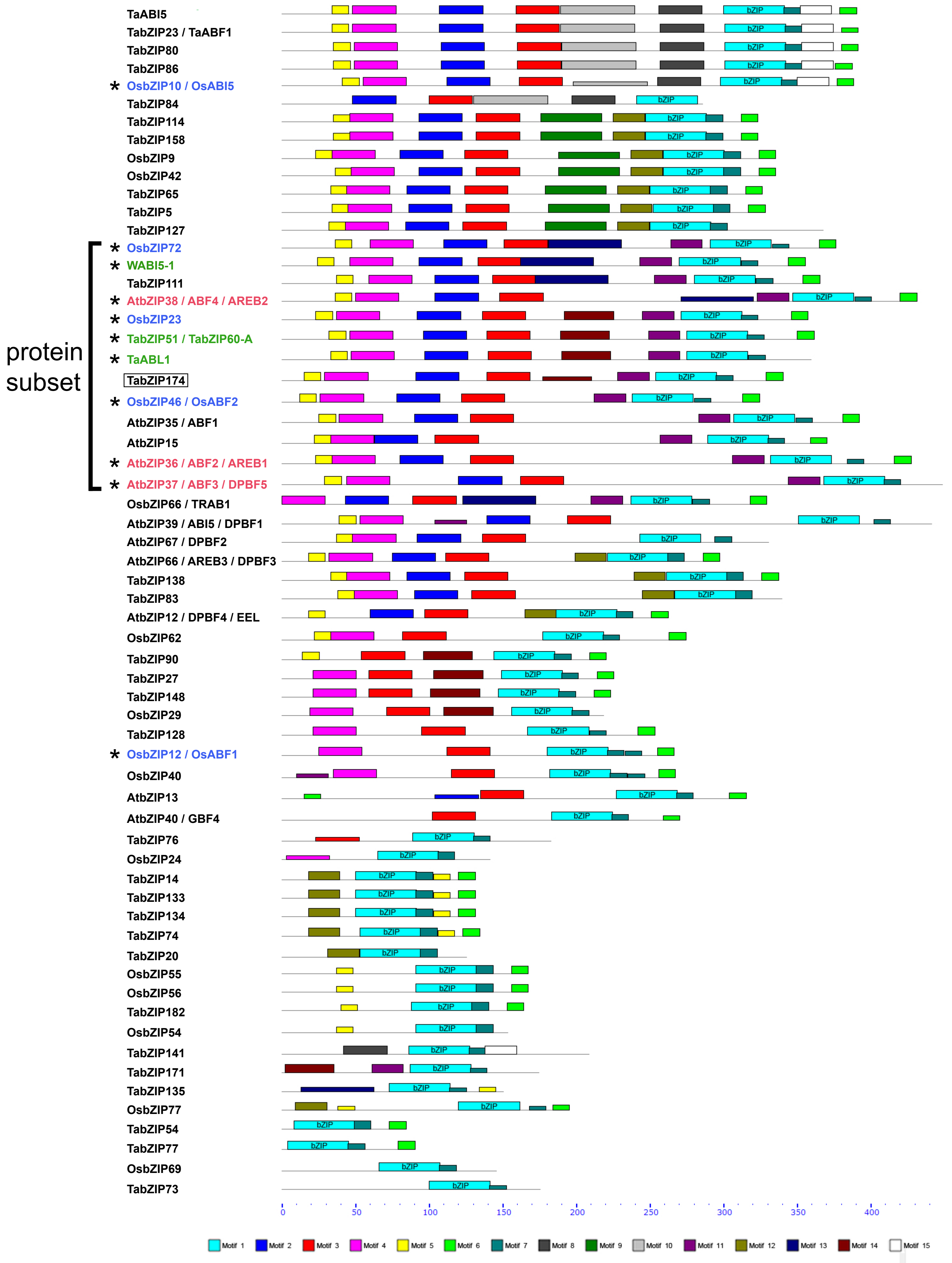

Supplement: Supplementary file 1 [file Image1.PDF]
