# Supplementary material for: Bioinformatic Analyses of Subgroup-A Members of the Wheat bZIP Transcription Factor Family and Functional Identification of TabZIP174 Involved in Drought Stress Response
Source: Front Plant Sci. 2016 Nov 16;7:1643. doi: 10.3389/fpls.2016.01643 (PMC5110565; doi:10.3389/fpls.2016.01643)
Supplement: Supplementary file 2 [file Image2.PDF]

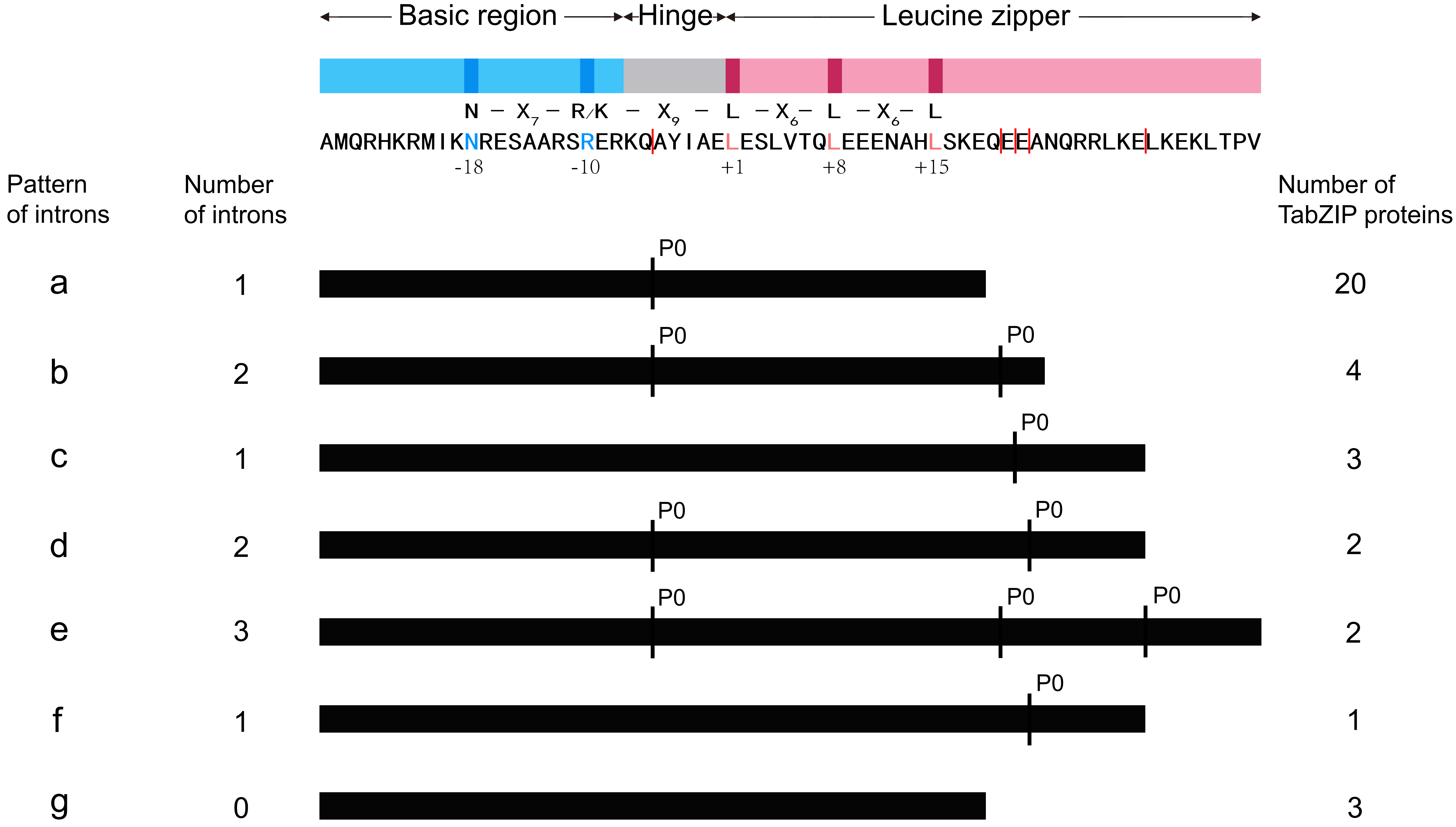

Supplementary Figure 2. Distribution and phases of introns within the bZIP domains of 35 novel Subgroup-A TabZIP proteins
The 35 Subgroup-A *TabZIP* genes are divided into seven patterns (a~g), based on intron numbers and positions. The amino acid residues of the bZIP domain region in an example sequence are shown at the top, and the colored letters represent highly conserved amino acid residues in this bZIP domain. The first leucine (L) residue in the leucine zipper region is marked with +1, and upstream and downstream amino acid residues of this leucine residue are marked with negative and positive numbers, respectively. Black bars below, representing the bZIP domains of the TabZIP proteins, are drawn to scale based on the actual lengths of corresponding bZIP domains. Red vertical lines inserted between amino acid residues of the example sequence and black vertical lines within the bZIP domains indicate the intron positions. P0 (Phase 0) refers to the splicing phase of the intron within the bZIP domain. The number of introns and the number of TabZIP proteins in each pattern are also displayed.
