# Supplementary material for: Bioinformatic Analyses of Subgroup-A Members of the Wheat bZIP Transcription Factor Family and Functional Identification of TabZIP174 Involved in Drought Stress Response
Source: Front Plant Sci. 2016 Nov 16;7:1643. doi: 10.3389/fpls.2016.01643 (PMC5110565; doi:10.3389/fpls.2016.01643)
Supplement: Supplementary file 3 [file Image3.PDF]

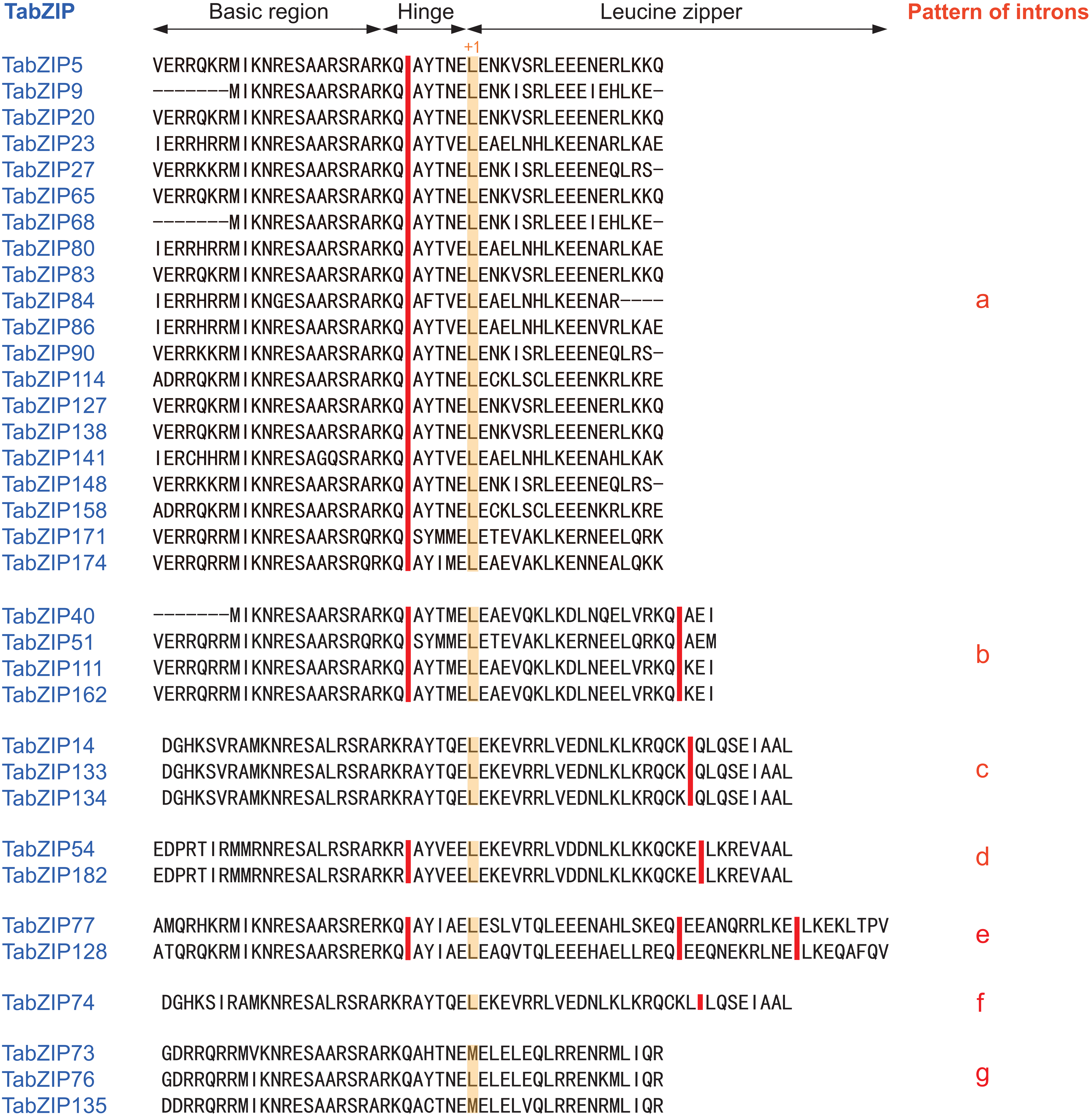

Supplementary Figure 3. Positions and patterns of introns within the bZIP domains of 35 novel Subgroup-A TabZIP proteins

Red lines indicate the positions of the introns inserted in the bZIP domains. All of the introns are in Phase 0 (P0). The first leucine residue in the leucine zipper region is marked with +1 as a position reference.
