# Supplementary material for: Bioinformatic Analyses of Subgroup-A Members of the Wheat bZIP Transcription Factor Family and Functional Identification of TabZIP174 Involved in Drought Stress Response
Source: Front Plant Sci. 2016 Nov 16;7:1643. doi: 10.3389/fpls.2016.01643 (PMC5110565; doi:10.3389/fpls.2016.01643)
Supplement: Supplementary file 4 [file Image4.PDF]

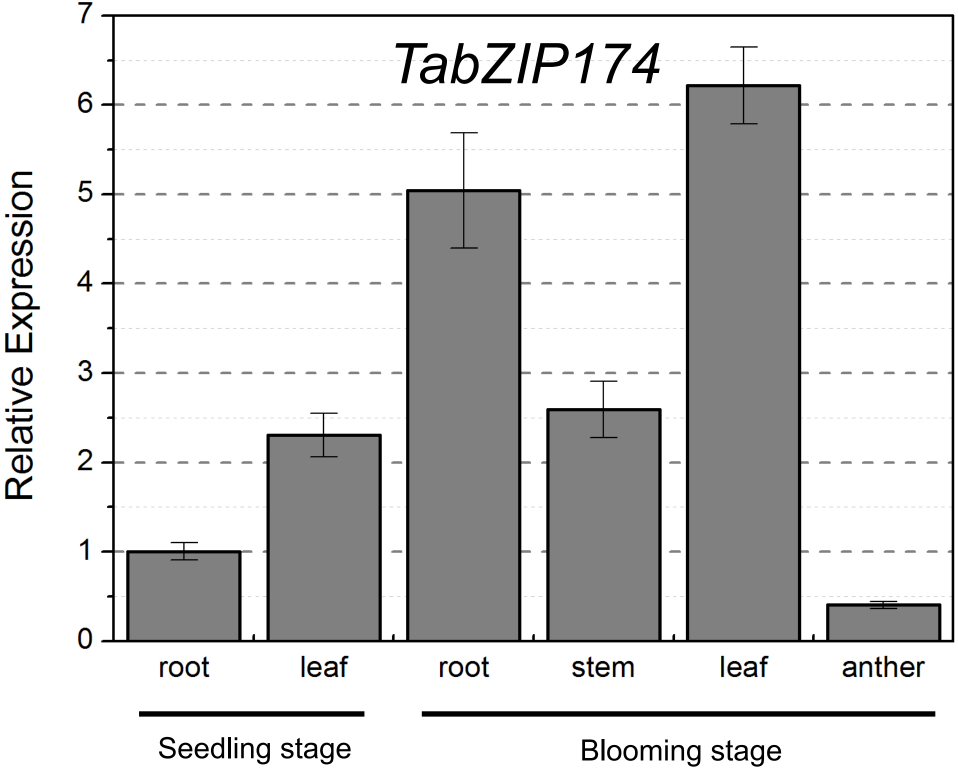

Supplementary Figure 4. Expression pattern of *TabZIP174* in wheat tissues at the seedling and blooming stages

The $2^{-\Delta\Delta C_T}$ method was used to calculate the relative expression levels of *TabZIP174* in different tissues. The expression of *TabZIP174* in seedling root is regarded as a reference, and other values represent the expression levels relative to the reference. Mean values and SDs were obtained from three biological replicates.
